# Advanced glycation end products dietary restriction effects on bacterial gut microbiota in peritoneal dialysis patients; a randomized open label controlled trial

**DOI:** 10.1371/journal.pone.0184789

**Published:** 2017-09-20

**Authors:** Rabi Yacoub, Melinda Nugent, Weijin Cai, Girish N. Nadkarni, Lee D. Chaves, Sham Abyad, Amanda M. Honan, Shruthi A. Thomas, Wei Zheng, Sujith A. Valiyaparambil, Mark A. Bryniarski, Yijun Sun, Michael Buck, Robert J. Genco, Richard J. Quigg, John C. He, Jaime Uribarri

**Affiliations:** 1 Department of Internal Medicine, Jacobs School of Medicine and Biomedical Sciences, University at Buffalo, Buffalo, New York, United States of America; 2 Department of Internal Medicine, Icahn School of Medicine at Mount Sinai, New York, New York, United States of America; 3 Department of Computer Science and Engineering, University at Buffalo, Buffalo, New York, United States of America; 4 Department of Biochemistry, Jacobs School of Medicine and Biomedical Sciences, University at Buffalo, Buffalo, New York, United States of America; 5 Department of Phamaceutical Sciences, University at Buffalo School of Pharmacy and Pharmaceutical Sciences, Buffalo, New York, United States of America; 6 Department of Microbiology and Immunology, Jacobs School of Medicine and Biomedical Sciences, University at Buffalo, Buffalo, New York, United States of America; 7 Department of Oral Biology, Jacobs School of Medicine and Biomedical Sciences, University at Buffalo, Buffalo, New York, United States of America; Universidade Estadual Paulista Julio de Mesquita Filho, BRAZIL

## Abstract

The modern Western diet is rich in advanced glycation end products (AGEs). We have previously shown an association between dietary AGEs and markers of inflammation and oxidative stress in a population of end stage renal disease (ESRD) patients undergoing peritoneal dialysis (PD). In the current pilot study we explored the effects of dietary AGEs on the gut bacterial microbiota composition in similar patients. AGEs play an important role in the development and progression of cardiovascular (CVD) disease. Plasma concentrations of different bacterial products have been shown to predict the risk of incident major adverse CVD events independently of traditional CVD risk factors, and experimental animal models indicates a possible role AGEs might have on the gut microbiota population. In this pilot randomized open label controlled trial, twenty PD patients habitually consuming a high AGE diet were recruited and randomized into either continuing the same diet (HAGE, *n* = 10) or a one-month dietary AGE restriction (LAGE, *n* = 10). Blood and stool samples were collected at baseline and after intervention. Variable regions V3-V4 of 16s rDNA were sequenced and taxa was identified on the phyla, genus, and species levels. Dietary AGE restriction resulted in a significant decrease in serum N^*ε*^-(carboxymethyl) lysine (CML) and methylglyoxal-derivatives (MG). At baseline, our total cohort exhibited a lower relative abundance of *Bacteroides* and *Alistipes* genus and a higher abundance of *Prevotella* genus when compared to the published data of healthy population. Dietary AGE restriction altered the bacterial gut microbiota with a significant reduction in *Prevotella copri* and *Bifidobacterium animalis* relative abundance and increased *Alistipes indistinctus*, *Clostridium citroniae*, *Clostridium hathewayi*, and *Ruminococcus gauvreauii* relative abundance. We show in this pilot study significant microbiota differences in peritoneal dialysis patients’ population, as well as the effects of dietary AGEs on gut microbiota, which might play a role in the increased cardiovascular events in this population and warrants further studies.

## Introduction

Advanced glycation end products (AGEs) are a heterogeneous group of compounds that are formed by the non-enzymatic reaction of reducing α-carbonylic compounds with free amino groups in proteins, lipids and nucleic acids resulting in alteration in their function [1–3]. We know, however, that AGEs may form through many other biological pathways [4]. AGEs are constantly formed at a small rate endogenously and a significant amount is consumed as food. There is significant literature linking elevated AGE levels with increased cardiovascular risk [5, 6], diabetes [7, 8], aging [9, 10], and renal dysfunction [11–14]. AGE accumulation can be harmful through several mechanisms including affecting protein structure and function by directly cross-linking with them, or by activating cellular receptors [15–18].

In subjects with normal renal function, AGEs are excreted in urine. However, in patients with end stage renal disease (ESRD) requiring hemodialysis (HD) or peritoneal dialysis (PD), AGEs build up in the body due to impaired urinary elimination and limited clearance during dialysis [19–21].

Due to food preparation methods, modern western diets contain large amounts of AGEs [22, 23], and the estimated daily amount supplied ranges from 25 to 75 mg of mainly pyrraline and carboxymethyllysine (CML) [24]. It has been suggested that about 10–30% of the ingested load of dietary AGEs (dAGEs) gets absorbed into the body and becomes incorporated in the body AGE pool [25–27]. The remainder of the ingested AGEs travels to the colon where it could interact with the bacterial microbiota [28–30]. Methylglyoxal (MG) is a reactive dicarbonyl intermediate and an AGE precursor [31]. Its concentration is increased in diabetes and it is known to increase oxidative stress [31], worsen vascular damage, and promote atherosclerosis and cardiovascular events [32, 33]. MG can lead to increase AGE induction resulting in worsening oxidative stress [31], diabetic myopathy [7], chronic low-grade inflammation, and impaired extracellular matrix remodeling [34].

*In vitro* studies have indicated that Maillard reaction products (MRPs) may affect bacterial growth [35] and gut microbiota composition, and that micro-organisms can degrade AGEs [35, 36]. Anaerobic bacteria, particularly *Bifidobacteria* strains, have been shown to be able to use bread melanoidins (the final products of the Maillard reaction) as a carbon source [29]. This indicates that dAGEs might affect gut microbiota through negative selection (direct toxic effects), or positive selection (favoring bacterial species overgrowth that can utilize dAGEs as source of energy). In this pilot study, we aimed to evaluate the effect of restricting a habitually high dAGE consumption on gut microbiota in a group of ESRD patients on maintenance PD, and hypothesize that dAGE restriction affects the diversity of bacterial gut microbiota in patients with ESRD receiving maintenance PD.

## Materials and methods

### Study participants and enrollment

**Fig 1:**
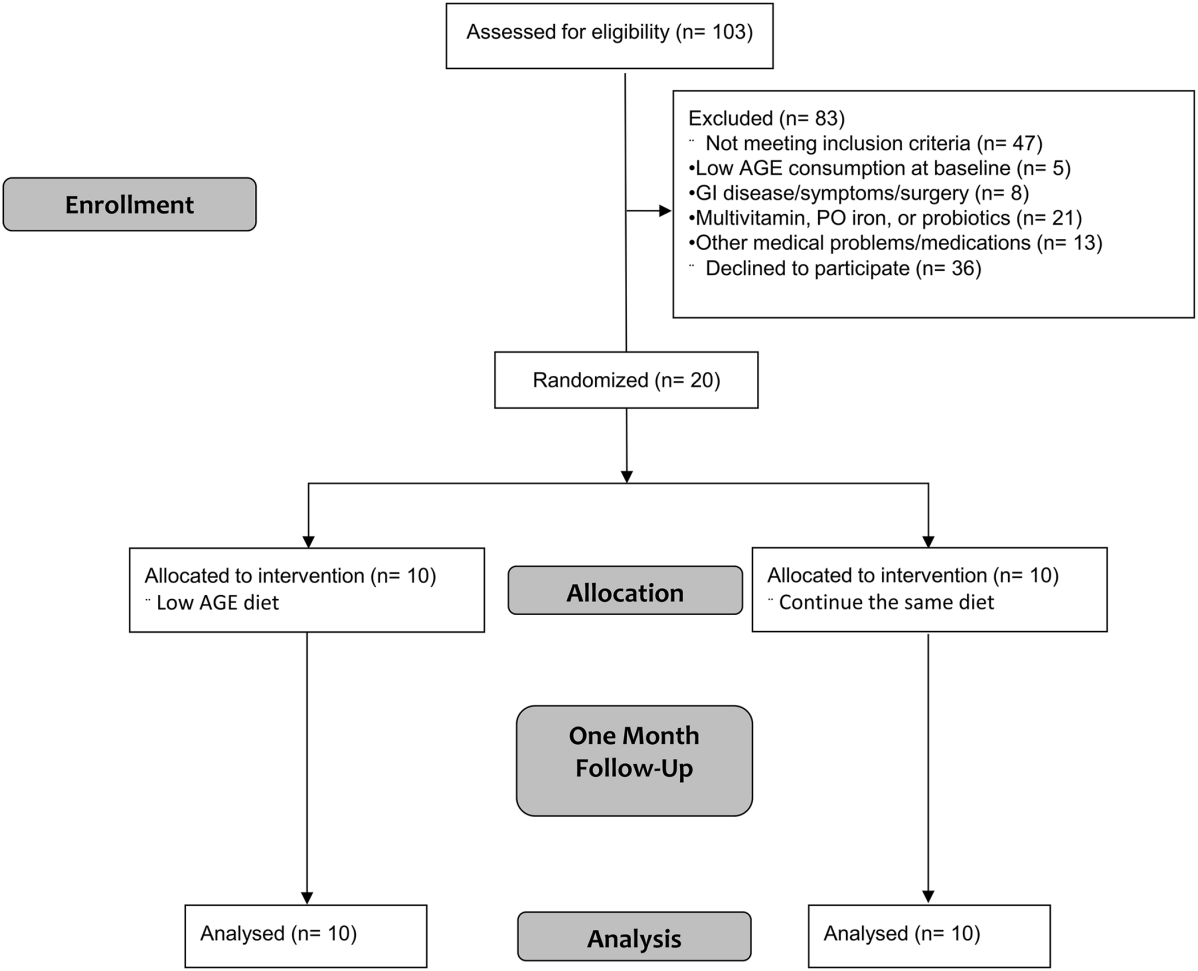
CONSORT flowchart. After enrollment, patients were randomized to either continue consuming high AGE diet, or a one month dAGE restriction. Stool and blood samples were collected at baseline and after intervention. AGEs, Advanced glycation end products; GI, gastrointestinal; PO, per os.

This is a randomized, open label trial. This pilot study was aimed at exploring the effects of dAGEs restriction on the bacterial gut microbiota composition in PD patients. Patients were recruited from the Icahn School of Medicine at Mount Sinai PD unit from June 2015 until December 2015 (ClinicalTrials.gov; NCT02467530). Screening and feasibility evaluation was initiated on January 2015, and study protocol (Study protocol) was submitted on June 8^th^ 2015. The first patient was recruited after finalizing the study submission on ClinicalTrials.gov. The authors confirm that all ongoing and related trials for this intervention are registered. All patients signed informed consent; the trial was conducted in accordance with the Declaration of Helsinki and good clinical practice guidelines. All research involving human participants was approved by the Icahn School of Medicine at Mount Sinai Institutional Review Board (IRB, GCO 14–1961, first approved December, 2^nd^ 2014 till December, 1^st^ 2015), and all clinical investigation have been conducted according to the principles expressed in the declaration of Helsinki. Written informed consent was obtained before enrolling. Inclusion criteria for the study included patients with ESRD receiving PD for at least 2 months, 18 years of age or older who consumed a diet high in AGEs (>12 Eq/day) [23]. Patients with advanced liver disease, heart failure, autoimmune disease, and those receiving probiotics, immunosuppressant medications, steroids, chemotherapy, multivitamins, or oral iron supplements were excluded. Patients who received a course of antibiotics were eligible three months after the last dose. Patients with medical history of any cancer, abdominal surgery, or bowel obstruction and those who sustained lower gastrointestinal bleeding were excluded (Fig 1, Consort 2010 checklist). No changes to methods after trial commencement were implemented.

### Randomization and intervention protocol

Pre-prepared opaque envelopes were used to randomize subjects. After signing the consent, patients were randomly given an envelope (by the principal investigator) with their allocation, which was opened immediately to reveal their assignment. Patients had an initial interview with the research dietitian to determine their habitual consumption of a high AGE diet and then randomized to either a high (HAGE) or low AGE (LAGE) diet. Participants randomized to continue the same high AGE (HAGE) diet were instructed to continue eating as usual for the next one month and simply record a three-day diet log just before each study visit, baseline, and one month. Those randomized to the low AGE (LAGE) were individually instructed on meal planning to meet study requirements while maintaining their usual peritoneal dialysis diet instructions. To vary the AGE content, foods, particularly meat, were exposed to different cooking methods. LAGE subjects were instructed to boil, poach, stew or steam, avoid fried entrees, and reheat food indirectly using steam in a double boiler. The subjects were followed closely by phone calls (1 to 2 times/week) to assure dietary compliance.

Medical treatment was not changed during this month; blood pressure medications, phosphate binders, and erythropoietin stimulating agents (ESA) doses were kept the same. No new antibiotics, probiotics, multivitamin, oral iron or antacid prescription was initiated during this period. All participants’ samples and data were labeled using new unique identifiers and procedures and measurements were all conducted blindly.

### Stool sample collection and DNA extraction

Stool samples were collected by the participants at home on the morning of both visits, placed in sterile, DNase, RNase and pyrogens free 50 ml tubes, transported on ice, and stored in -80 C within four hours of defecating. DNA was extracted using PowerFecal^®^ DNA isolation kit, according to the manufacturer protocol (MOBIO laboratory Inc. QIAGEN Company CA. USA). Two separate isolations from each sample were obtained from a grossly firm stool area absent of undigested food, and placed immediately in extraction tubes. DNA extraction was performed in duplicates on each sample and only samples with DNA concentration ≥10 ng/microliter were sequenced.

### 16s sequencing and annotation

Metagenomic DNA was amplified using the 16S V3 (341F) forward and V4 (805R) reverse primer pairs with Illumina adapter overhang. Reactions were purified using Agencourt AMPure XP beads (Beckman Coulter). Illumina sequencing adapters and dual Indexes were added to the amplified fragments using an 8 cycle PCR reaction, further purified as above, and quantified using Quant-iT PicoGreen assay (Invitrogen). Quality control, quantification and average size distribution of the libraries was assessed with the Advance Analytical Fragment Analyzer. Libraries were normalized and pooled to 4 ηM, further quantified using NEBNext NGS library quantification kit (NEB) prior to being denatured and diluted to a final concentration of 10 pM with a 20% PhiX spike-in (Illumina) control. Sequencing was performed using MiSeq Reagent V3 Kit (illumina) using 2 × 300 bp paired-end sequencing.

For the OTU picking, we merged Illumina paired-end reads using Pear (version 0.9.6)[37] with default parameters applied. To improve taxonomic accuracy, we applied strict sequence quality filtering. A sequence read with 90% of base quality scores higher than or equal to Q30 was considered as good quality read. After trimming the forward and reverse primer, we used UPARSE (version 8.0)[38] to perform *de novo* OTU picking at different distance levels ranging from 0.03 to 0.20 with step size 0.01. We removed chimera sequences by searching against the gold UCHIME reference (http://drive5.com/uchime/gold.fa). By using QIIME (version 1.9.1)[39] command line, the species level OTUs defined at the 0.03 distance level were annotated by searching the representative sequence of each OTU against the Greengenes database (version 13.5) [40].

### Measurements

#### Dietary intake

Assessment of daily dAGE content was based on 3-day food records and estimated from a database of ∼560 foods that lists AGE values expressed as AGE equivalents per day (1 AGE equivalent = 1000 kilo units). Nutrient intakes were estimated from food records using a nutrient software program (Food Processor, version 10.1; ESHA Research, Salem, OR, USA).

#### AGE determination

AGEs (CML and MG) in serum were determined by well-validated, competitive ELISAs based on monoclonal antibodies for protein-bound CML (4G9) and protein-bound MG derivatives, i.e. arginine-MG-H1, characterized by HPLC [4, 23, 25–27]. We initially aimed to determine AGE concentration using skin fluorescent AGE measurements. Since not all AGEs exhibit fluorescent properties, we decided to use more precise ELISA-based test [41–43].

### Statistical analysis

Descriptive analyses summarized continuous variables at baseline through their mean (SD) and median (first and third quartiles). Categorical variables were summarized using percentages. AGE levels were reported as time point values and the difference between post intervention and baseline concentrations. This is a pilot study to evaluate the effects of dAGE restriction on gut microbiota in PD patients; no previous similar study was conducted to aid in evaluating and assessing sample size needed and power analysis. We believe that this current study will provide this information for future studies.

## Results

### Patient characteristics

Patients were recruited until we reached the goal of 10 participants in each group. Of the 103 ESRD patients initially screened, five were excluded because of a low AGE diet at baseline. There was no significant difference between the two groups in regards to age, gender or race (Table 1). All participants were receiving PD at Mount Sinai Hospital for more than 2 months, with similar dialysis vintage between the two groups, and no statistically significant difference in regards to use of phosphate binders or erythropoietin. Laboratory data at baseline including baseline AGE levels CML and MG were similar, except for lower ferritin levels in the HAGE group (Tables 1 and 2). As expected, the low AGE diet group showed a significant decrease in serum AGE levels post intervention (Table 2).

**Table 1:** Patient basic characteristics.

Characteristics	HAGE (N = 10)	LAGE (N = 10)	P*
Age (years)^≠^	50.6 ± 16.2	49.7 ± 11.4	0.8
Weight (kg) ^≠^	78.1 ± 12.3	78.8 ± 13.8	0.9
BMI (Baseline) ^≠^	27.5 ± 4.1	25.8 ± 3.7	0.3
BMI (after intervention) ^≠^	27.13±3.6	25.73±4.3	0.4
Systolic Bp (mmHg) ^≠^	142 ± 23	136 ± 24	0.6
Diastolic Bp (mmHg) ^≠^	83 ± 16	83 ± 13	0.9
Diabetes Mellitus	1	2	0.9
Gender (N)			
Male	4	6	0.7
Female	6	4	
Race (N)			
African Americans	4	6	0.5
White	3	3	
Hispanics and others	3	1	
Dialysis procedure and membrane			
Icodextrin use	2	5	0.3
>twice daily D2.5% solution	4	5	0.9
Kt/v^≠^	2.3 ± 0.58	2.43 ± 0.81	0.7
APD/CAPD	4/6	4/6	0.9
Dialysis Vintage (weeks)^≠^	97 ± 154	132 ± 113	0.6
Baseline Diet			
Protein (gr/day) ^≠^	87.5 ± 19.4	84.69 ± 20.7	0.8
Fat (gr/day) ^≠^	83.45 ± 29.9	81.54 ± 32.9	0.9
Carbohydrates (gr/day) ^≠^	257.17 ± 41.9	219.92 ± 72.1	0.3
Fiber (gr/day) ^≠^	19.27 ± 3.5	13.87 ± 5.2	0.06
Sugar (gr/day) ^≠^	94.22 ± 59.1	83 ± 36.6	0.6
Calories^≠^	2123.78 ± 464.9	1828.38 ± 439.8	0.3
Baseline laboratory data			
iPTH (pg/ml)	488±673	287±107	0.4
Fe (mcg/dl)	82.3 ± 23.3	61.9 ± 22.4	0.08
Tsat (%)	29.4 ± 8.1	28.3 ± 7.9	0.8
Ferritin (ng/ml)	254.4 ± 202.8	579.3 ± 248.5	0.01
Hgb (g/dl)	9.86 ± 2.1	9.58 ± 1.5	0.7
Medications			
Phosphate binders	6	7	0.9
ESA	6	9	0.3

*Comparisons between the LAGE and HAGE groups is considered to be statistically significant at P<0.05 level (two tailed Chi-Square, Fisher exact and Mann-Whitney U test when applicable).

^≠^ Mean ± standard deviation. APD/CAPD: Automated peritoneal dialysis/Continuous ambulatory peritoneal dialysis.

**Table 2:** AGE levels before and after intervention.

AGE levels	HAGE (N = 10)	LAGE (N = 10)	*P
Baseline AGE levels			
CML (unit/mL) ^≠^	26.96 ± 2.9	26.18 ± 6.5	0.7
MG (nmol/mL) ^≠^	5.3 ± 1.7	4.79 ± 1.6	0.5
Post intervention AGE levels			
CML (unit/mL) ^≠^	29.59 ± 4.6	23.29 ± 4.3	0.004
MG (nmol/mL) ^≠^	5.61 ± 1.3	4 ± 1.2	0.009
Delta AGEs (Changes from baseline)			
CML (unit/mL) ^≠^	2.89 ± 4.1	-2.64 ± 3.5	0.004
MG (nmol/mL) ^≠^	0.79 ± 1.2	-0.31 ± 0.9	0.027

*Comparisons between the LAGE and HAGE groups is considered to be statistically significant at P<0.05 level (Mann-Whitney U test).

^≠^ Mean ± standard deviation. HAGE, high advanced glycation end products group; LAGE, low advanced glycation end products group; CML, N^*ε*^-(carboxymethyl) lysine; MG, methylglyoxal.

### Baseline gut microbiota profile in ESRD patients on PD

To better understand the compositions of bacterial gut microbiota in ESRD patients on PD, we first performed a descriptive analysis to evaluate the relative abundance at phyla, genus, and species levels. In our cohort, *Bacteroidetes* and *Firmicutes* accounted for approximately 56% and 32%, respectively of the phyla (S1A Fig, S1 Table). At the genus level, we found that *Bacteroides* and *Prevotella* both accounted for 50%; *Faecalibacterium*, *Blautia*, *Ruminococcus*, *Akkermansia*, *Parabacteroides*, *Roseburia*, Esc*h*erichia, and *Clostridium* accounted for 40%; and the rest of 66 genus accounted for less than 10% of the gut bacterial relative abundance (S1B Fig, S2 Table). Bacterial species that showed high relative abundance (>5%) in our cohort at baseline were: *Prevotella copri*, *Faecalibacterium prausnitzii*, *Bacteroides ovatus*, *Bacteroides fragilis*, and *Bacteroides uniformis* (S3 Table). Our data show high variability across the subjects within each group in regards to species identified, suggesting multiple environmental/co-morbid conditions beyond dietary AGE consumption.

### Dietary AGE modification effects on gut microbiota

**Fig 2:**
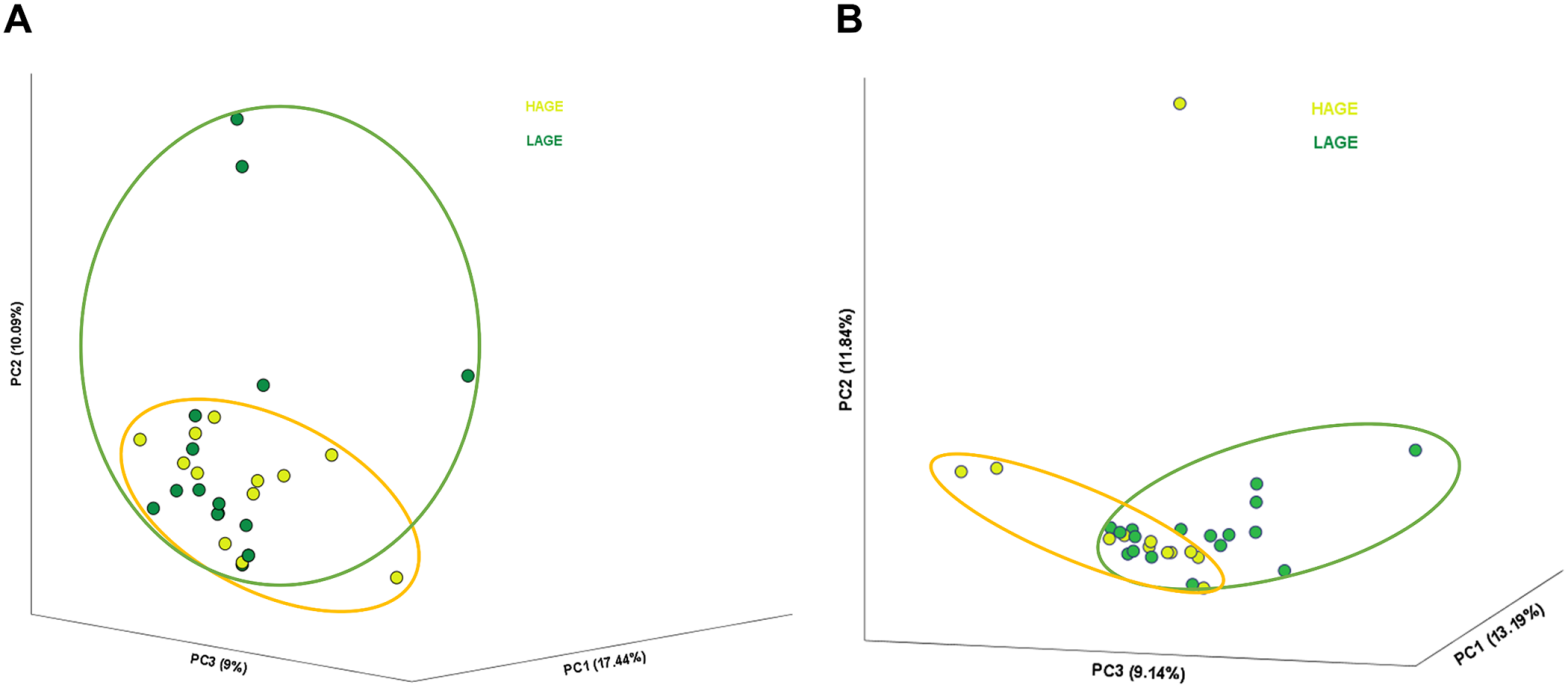
One month dAGE restriction resulted in changes in gut bacterial microbiota. Operational taxonomic units were annotated and analyzed at species levels, and shows no difference in microbiota projection onto the principal components at baseline (A), followed by changes after dietary intervention (B). HAGE, high advanced glycation end products group; LAGE, low advanced glycation end products group.

**Fig 3:**
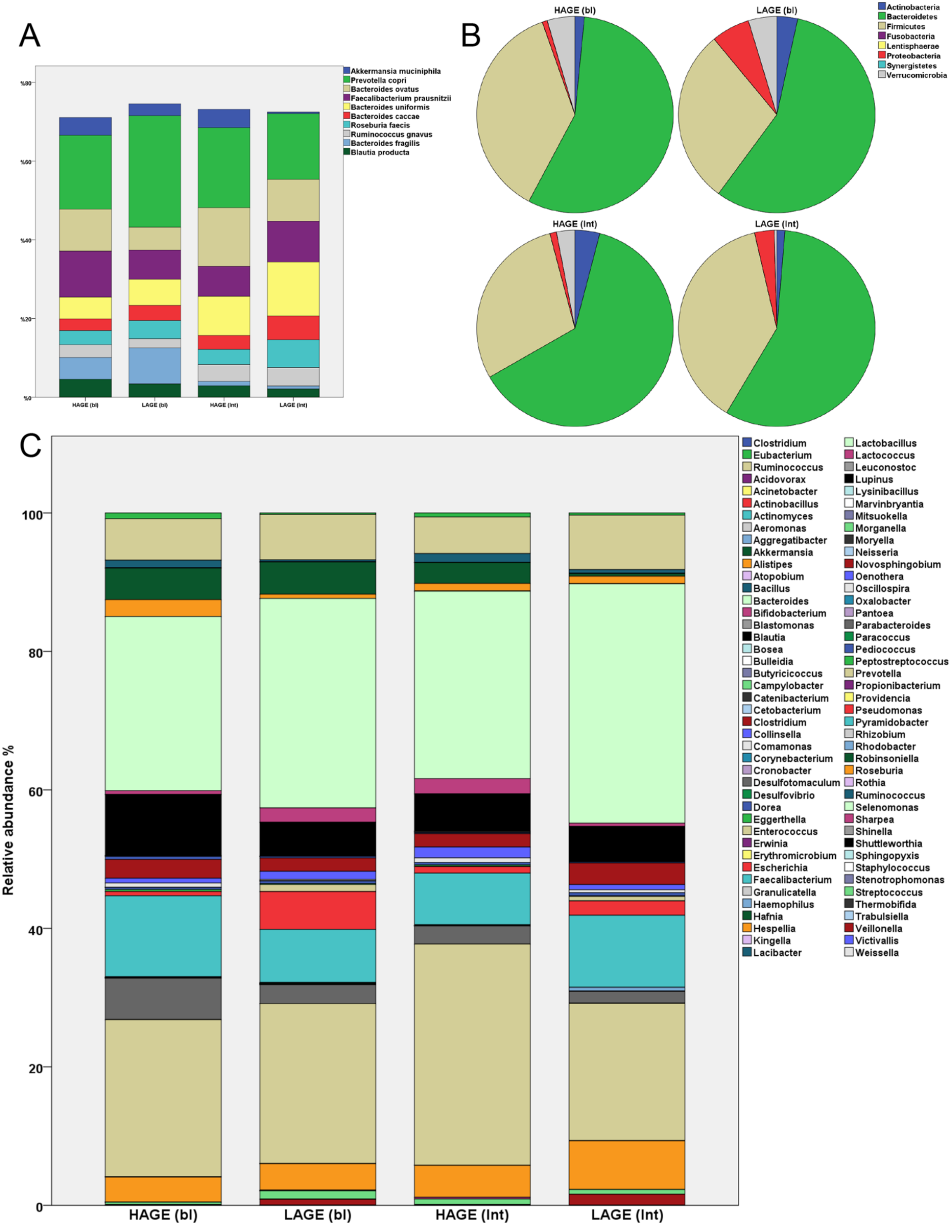
Species (A), genus (B), and Phyla (C) differences between groups at baseline and after intervention. HAGE (bl), high advanced glycation end products group at baseline; LAGE (bl), low advanced glycation end products group at baseline; HAGE (Int), high advanced glycation end products group after intervention; LAGE (Int), low advanced glycation end products group after intervention. The top ten species in relative abundance (74%) are shown in Fig 3A, full species are shown in S2 Fig.

**Fig 4:**
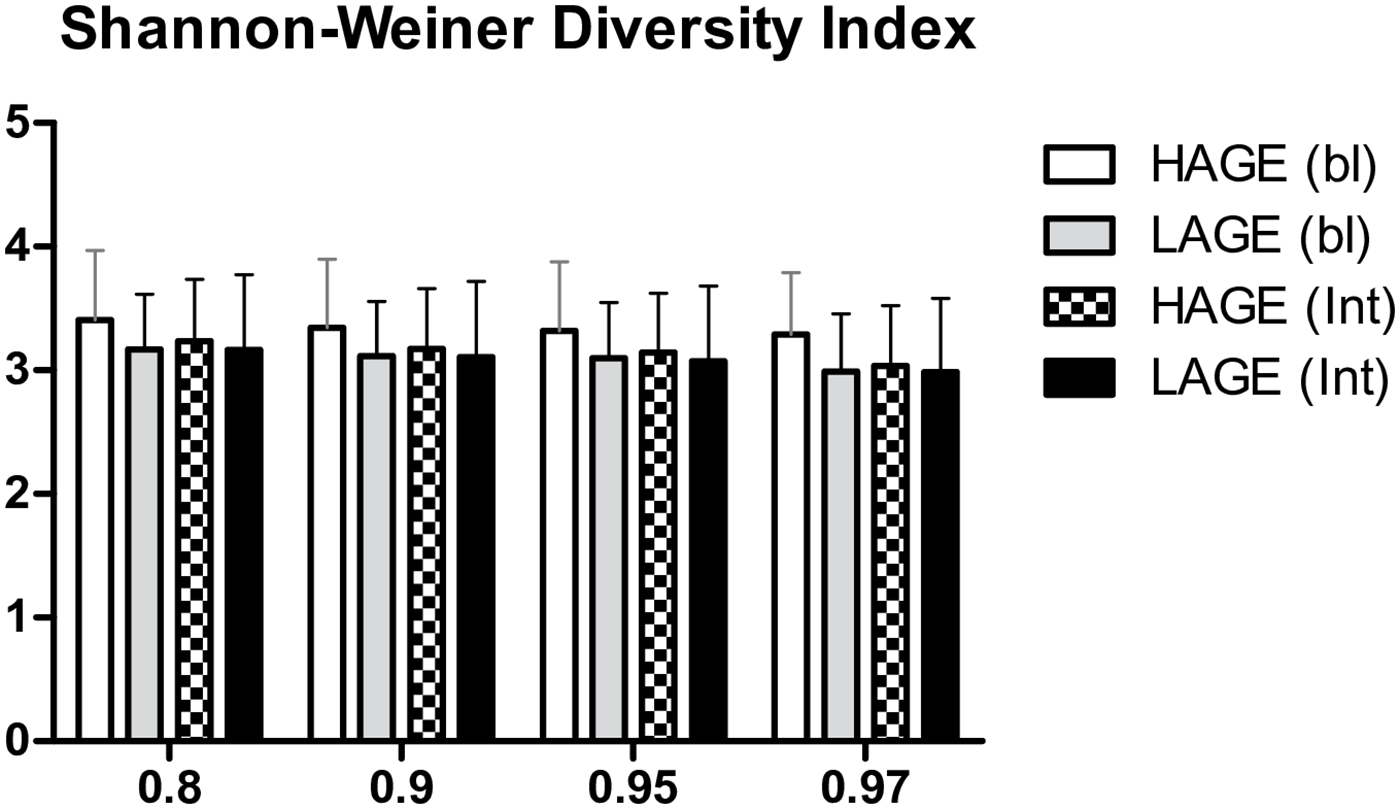
Shannon-Wiener diversity index indicating no differences among groups before and after intervention (bars and columns represent diversity index mean ± standard deviation). HAGE (bl), high advanced glycation end products group at baseline; LAGE (bl), low advanced glycation end products group at baseline; HAGE (Int), high advanced glycation end products group after intervention; LAGE (Int), low advanced glycation end products group after intervention.

The gut microbiota composition was studied separately at both baseline and post intervention. At baseline, dimension reduction analysis showed that the three leading principal components (PC) accounted for 17.44%, 10.09%, and 9% of variances, respectively, and there was no difference in the gut microbiota relative abundance between the two groups (Fig 2A). After intervention, dimension reduction resulted in three leading PCs explaining 13.19%, 11.84%, and 9.14% of variances, respectively. The two groups started showing small distinction in their projection onto the three principal components (Fig 2B). We found that the distinction between the two groups is mostly due to their projection onto the third principal component (*P* = 0.047), while subjects’ projection onto the first and second principal component were not statistically significant (*P* = 0.345, and *P* = 0.308 respectively). Analysis of the bacterial species that have contributed significantly in the third principal component (and all components) is shown in S4 Table. It is important to note that in our analysis we have ignored small coefficients (absolute factor coefficient <0.3) to make sure that the bacterial species identified are the ones with the most effects. From the table we notice that most of the bacterial species have positively or negatively influenced only one of the principal components. This indicates that these species increase/decrease in relative abundance in a dependent fashion in our patient population. This is of certain importance as when each species relative abundance was tested and compared between the two groups, a limited set of bacteria have shown a significant relative abundance differences after changing AGE dietary habits. (Table 3, Fig 3A, S2 Fig). Comparing the bacterial species that changed in relative abundance from baseline after intervention (delta OTUs in Table 3) to their participation in each PC, reveals that *Prevotella copri*, *Clostridium citroniae*, *and Clostridium hathewayi* contributed solely to PC3, while *Ruminococcus gauvreauii* contributed to only PC2, and *Bifidobacterium animalis* and *Alistipes indistinctus* have not contributed to more than 0.3 absolute coefficient to any of the components. Phylogenic analysis (Fig 3B) indicates an increase in *Firmicutes* abundance and a decrease in *Verrucomicrobia* in the dietary intervention group. Genus analysis shown in Fig 3C illustrates the changes observed between the groups, which did not reach statistical significance due to the small samples size. We then performed Shannon-Wiener diversity index test (Fig 4) on the unannotated operational taxonomic units (OTUs) at different similarity distances (80%, 90%, 95% and 97% that corresponds to phyla, class, genus, and species levels). No significant difference between all groups was found in diversity across different similarity distances.

**Table 3:** Species with significant differences between the groups.

Bacterial Species	Delta OTUs^≠^			Baseline^≠^			After intervention^≠^		
	HAGE (N = 10)	LAGE (N = 10)	P	HAGE (N = 10)	LAGE (N = 10)	P	HAGE (N = 10)	LAGE (N = 10)	P
*Bifidobacterium animalis*	0.001 ± 0.004	-0.002 ± 0.005	0.045	0.0018 ± 0.004	0.0028 ± 0.006	0.649	0.003 ± 0.007	0.0004 ± 0.001	0.182
*Prevotella copri*	9.69 ± 20.5	-7.19 ± 14.8	0.016	23.36 ± 24.61	25.89 ± 27.71	0.802	33.06 ± 32.2	18.69 ± 25.2	0.189
*Alistipes indistinctus*	-0.29 ± 0.57	0.018 ± 0.17	0.047	0.367 ± 0.679	0.064 ± 0.169	0.087	0.081 ± 0.175	0.082 ± 0.167	0.999
*Clostridium citroniae*	-0.441 ± 1.12	0.365 ± 0.78	0.031	0.782 ± 1.577	0.159 ± 0.233	0.118	0.341 ± 0.565	0.525 ± 0.817	0.505
*Clostridium hathewayi*	-1.194 ± 1.49	0.148 ± 1.04	0.008	1.982 ± 1.967	0.935 ± 1	.007	0.787 ±0.743	1.083 ±1.282	0.480
*Ruminococcus gauvreauii*	-0.046 ± 0.06	-0.002 ± 0.02	0.013	0.093 ± 0.126	0.014 ± 0.018	0.016	0.047 ± 0.084	0.011 ± 0.018	0.101
*Solobacterium moorei*	-0.001 ± 0.001	0.0005 ± 0.002	0.017	0.001 ± 0.001	0.0001 ± 0.0004	0.016	0 ± 0	0.0005 ± 0.001	0.283
*Bryantella formatexigens*	-0.004 ± 0.008	-0.004 ± 0.012	0.966	0.02 ± 0.019	0.008 ± 0.015	0.100	0.013 ± 0.02	0.003 ± 0.007	0.019
*Catenibacterium mitsuokai*	0.154 ± 0.352	0.001 ± 0.006	0.083	0.071 ± 0.013	0.0007 ± 0.002	0.033	0.225 ± 0.43	0.002 ± 0.009	0.040

All Comparisons between the LAGE and HAGE groups is considered to be statistically significant at P<0.05 level (Mann-Whitney U test).

^≠^ Numbers reported as mean ± standard deviation. OUTs, operational taxonomic units; HAGE, high advanced glycation end products group; LAGE, low advanced glycation end products group.

## Discussion

This pilot study shows that a one-month intervention with dietary AGE restriction in a population of ESRD patients on maintenance PD resulted in changes in the composition of their gut microbiota. One of the biggest changes occurred in regards to *Prevotella copri* relative abundance, which significantly decreased following the low AGE diet. We confirmed previous work showing significant reduction in serum AGEs in response to a low AGE diet, which further indicates good adherence to the dietary instructions [44]. Moreover, compared to healthy participants from the human microbiome project (HMP) [45], our peritoneal dialysis patients appear to have unique different genera at baseline.

There are several lines of evidence to suggest gut microbiota is likely to be altered in patients with CKD [46]. In general, gut microbiota dysbiosis affects and increases the risk of multiple metabolic conditions including diabetes mellitus type 2, dyslipidemia, and obesity [47–54]. Numerous studies have shown the direct effects of alteration of gut bacterial compositions on traditional cardiac risk factors [55], and growing evidence in clinical studies suggests that obese individuals with insulin resistance are characterized by an altered composition of gut microbiota, particularly an elevated *Firmicutes*/*Bacteroidetes* ratio as compared with healthy people [56, 57].

Peritoneal dialysis solutions depend mostly on variable concentrations of dextrose [58, 59], and the high molecular weight glucose polymer icodextran to increase intraperitoneal tonicity and achieve adequate ultrafiltration [60]. Altered renal function, high prevalence of diabetes mellitus type 2, and the use of glucose based solutions all appear to play a role in the microbiota dysbiosis of ESRD PD patients. Our intervention and control groups were similar in almost all basic characteristics with the exception of baseline concentrations of serum ferritin. It is important to note that 50% of LAGE group patients were using icodextran compared to 20% of HAGE group. Though this could have affected the baseline bacterial composition between the two groups, no change in dialysis prescription was implemented during the study duration and OTUs changes from baseline in each patient were calculated to ensure normalization of these confounding factors.

We have performed a detailed descriptive analysis evaluating the composition of the gut microbiota in our cohort (S2 Table). Ding and Scholes divided the HMP participants based on their gut microbiota into four communities. Most of the four communities had a higher relative abundance of *Bacteroides* than our cohort [45]. The four healthy communities identified also showed much lower *Prevotella* abundance compared to our cohort. Interestingly, *Alistipes* accounted for less than 1.5% of the relative abundance in our cohort and was the 11^th^ genus in abundance compared to third in the HMP study [45], indicating that ESRD, the dialysis procedure, and/or the co-morbidities associated with it directly affect *Alistipes* abundance.

In general, *Prevotella* is considered a “good bacteria”. It uses xylan, xylose, and carboxymethylcellulose to produce high levels of short chain fatty acids (SCFAs) [61], while *Alistipes spp*. are pathogenic causing colitis and site specific tumors in animal studies [62]. The increased *Prevotella* and decreased *Alistipes* abundance in our cohort does not have to be very protective. *Prevotella copri* that accounted for almost 23% of all species relative abundances in our cohort has been found recently to be associated with autoimmune diseases, mainly rheumatoid arthritis [63, 64]. Detailed analysis of the baseline bacterial composition also reveals a very low percentage of *Bifidobacterium animalis* (*B*. *animalis*).

In healthy individuals, *B*. *animalis* accounts for almost 16% of the Bifidobacterial population [65], while in our cohort it accounted for less than 2%. Commercially, *B*. *animalis* is included in most of the probiotic treatments available as it is considered to confer beneficial effects.[65, 66] This decreased percentage of *B*. *animalis* compared to all *Bifidobacterium* may be of clinical importance and warrants further investigation. In our study, *B*. *animalis* is decreased in LAGE group and increased in HAGE, compatible with the previous in vitro studies [29, 35]. *Bifidobacterium* and *Lactobacillus* are also major lactate producing and pH regulating bacteria with the consumption of hexose sugars [67], thus altering intestinal sugar contents that would result in decreased available nutrients and affects their relative abundance.

Alpha diversity (the mean species diversity in each individual) did not seem to differ among the groups before and after intervention as shown in the Shannon-Weiner index test. However, small changes in the bacterial gut microbiota composition that might not be independently statistically significant on its own can add up to a significant shift in the overall bacterial composition between the groups when combined. As shown in Table 3 and S4 Table, significant bacterial taxas have contributed to the same principal component indicating a possible interaction among these taxas and the influence of the dietary AGEs on this interaction. The gut microbiota is dynamic and bacterial species interact closely either by competition (competing for the same nutrients) [68, 69], producing antibacterial substances to regulate other species, or by producing nutrients that can be used by other bacteria.

As shown in this evaluation, numerous subtle changes combined may result in a significantly different bacterial composition after intervention. Thus, it is of crucial importance to identify the correlation between all bacterial species residing in human gut. This interaction could of course be affected by diseases state (ESRD in this cohort). Understanding the interaction between these species will enable us to develop better and novel therapeutic strategies aimed at favoring the growth of the “good bacteria” at the expense of the “bad bacteria”. The correlation matrix in S5 Table explains the interaction between all species identified in the stool of our PD cohort.

In this report, we show in detail the bacterial gut microbiota changes associated with dAGE restriction. As shown before, AGE restriction plays a favorable role in cardio-protection and there is a strong association between certain bacterial metabolites/abundance and cardiovascular disease risk [11, 55]. Dietary AGE restriction may thus result in gut microbiota changes that could play a mechanistic role in the previously observed cardio-protection properties. This study represents a step towards addressing this and further experimental and population-based studies are warranted.

## Limitations and conclusion

Even though this study identified multiple bacterial species with altered relative abundance after dietary intervention, these findings should be read with caution. The human gut harbors numerous bacterial species, and this study is not powered to accurately identify the small changes in all species relative abundance changes. Due to the high variability and the small sample size, the effects of dialysis procedure, dialysate content or the renal failure itself on certain bacterial species needs to be further explored. Another issue is the comparison with published literature and the lack of normal control subjects-not on dialysis for direct comparison. The main goal of this study was to evaluate the effects of dietary AGE restriction on gut microbiota, and the unique microbiota found in total cohort compared with published normal subjects should be interpreted with caution. Though dAGEs decreased significantly in the intervention group it is hard to dissect the contribution of dAGE consumption effects on changes observed from the effects of modification in diet preparation. A potential limitation of the study is that despite measuring AGE serum levels at both baseline and after intervention, the initial assessment and inclusion procedures were based on estimated dietary intakes centered on dietary log and recall and these procedures are subjected to recall bias. However, we believe that the dietary assessment was reliable because dAGEs estimates were consistently high at both enrollment and first visit. Lastly, it is still possible that incomplete dietary adherence could have affected our results. To minimize lack of adherence, frequent nutritionist follow up phone calls were made to ensure strict adherence to assigned low/high AGE diets during the intervention. Moreover, results of serum AGE measurements before and after intervention suggest dietary compliance.

In summary, our findings again confirm that even short-term restriction of dAGE intake can significantly decrease circulating AGE levels in renal failure patients on maintenance peritoneal dialysis and further suggest that this dietary intervention may have an effect in the gut microbiota. Larger studies are needed to confirm the effect of dietary AGEs on the microbiota.

## Supporting information

S1 Fig Total cohort phylogenic and genus relative abundance. Figure A in S1: phyla level. Figure B in S1: genus level.
(TIFF)

S2 Fig Species differences between groups at baseline and after intervention. (TIF)

S1 Table Relative abundance of bacterial phyla at baseline. (DOCX)

S2 Table Relative abundance of bacterial genus at baseline. (DOCX)

S3 Table Relative abundance of bacterial species at baseline. (DOCX)

S4 Table Bacterial species principle components coefficients. (DOCX)

S5 Table Interaction between all species identified. (PDF)

S1 File Consort 2010 checklist: CONSORT 2010 checklist of information to include when reporting a randomized trial. (DOC)

S2 File Study protocol: Study protocol submitted for IRB approval. (DOC)
