# Supplementary figures and images for: Advanced glycation end products dietary restriction effects on bacterial gut microbiota in peritoneal dialysis patients; a randomized open label controlled trial
Source: PLoS One. 2017 Sep 20;12(9):e0184789. doi: 10.1371/journal.pone.0184789 (PMC5607175; doi:10.1371/journal.pone.0184789)

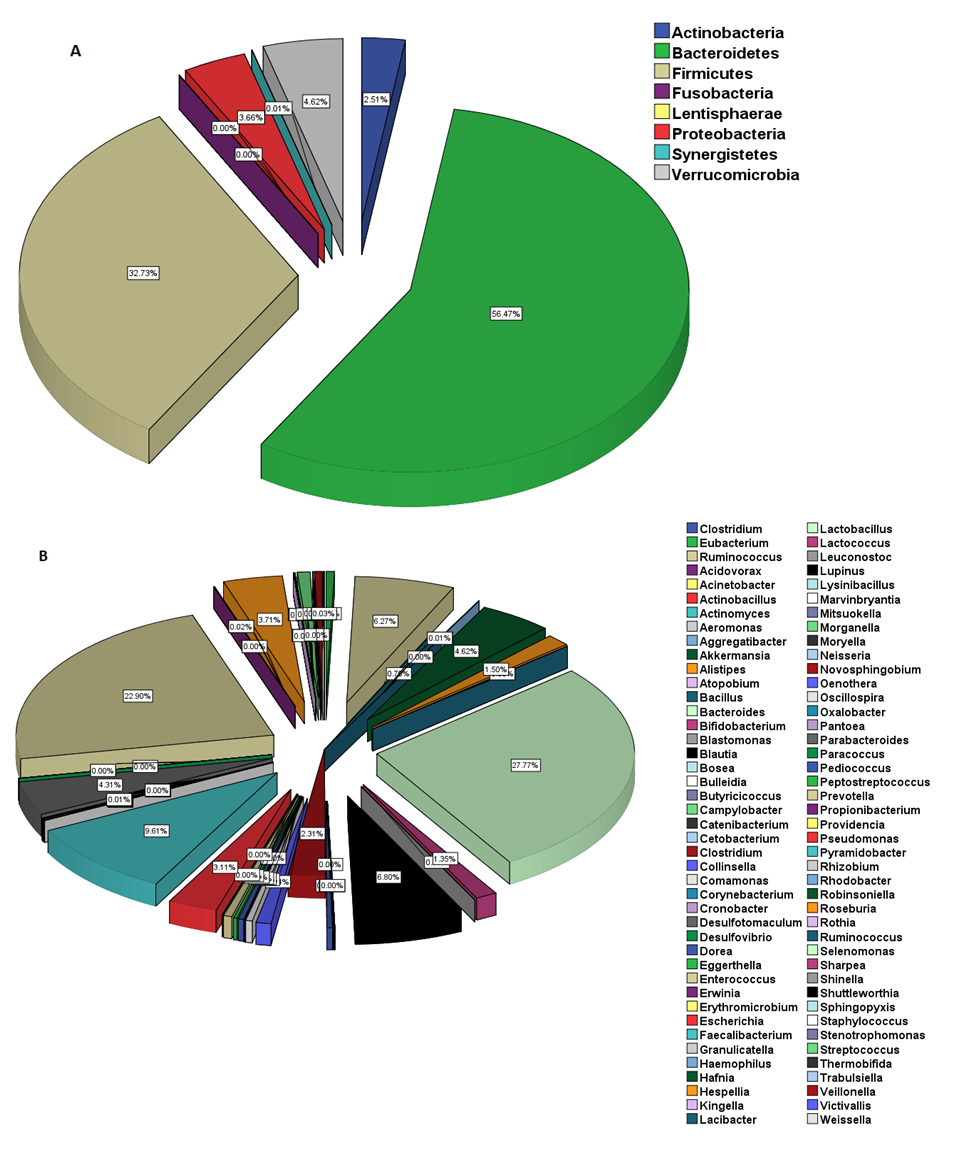

Supplement: S1 Fig — Figure A in S1: phyla level. Figure B in S1: genus level. (TIFF) [file pone.0184789.s001.tiff]

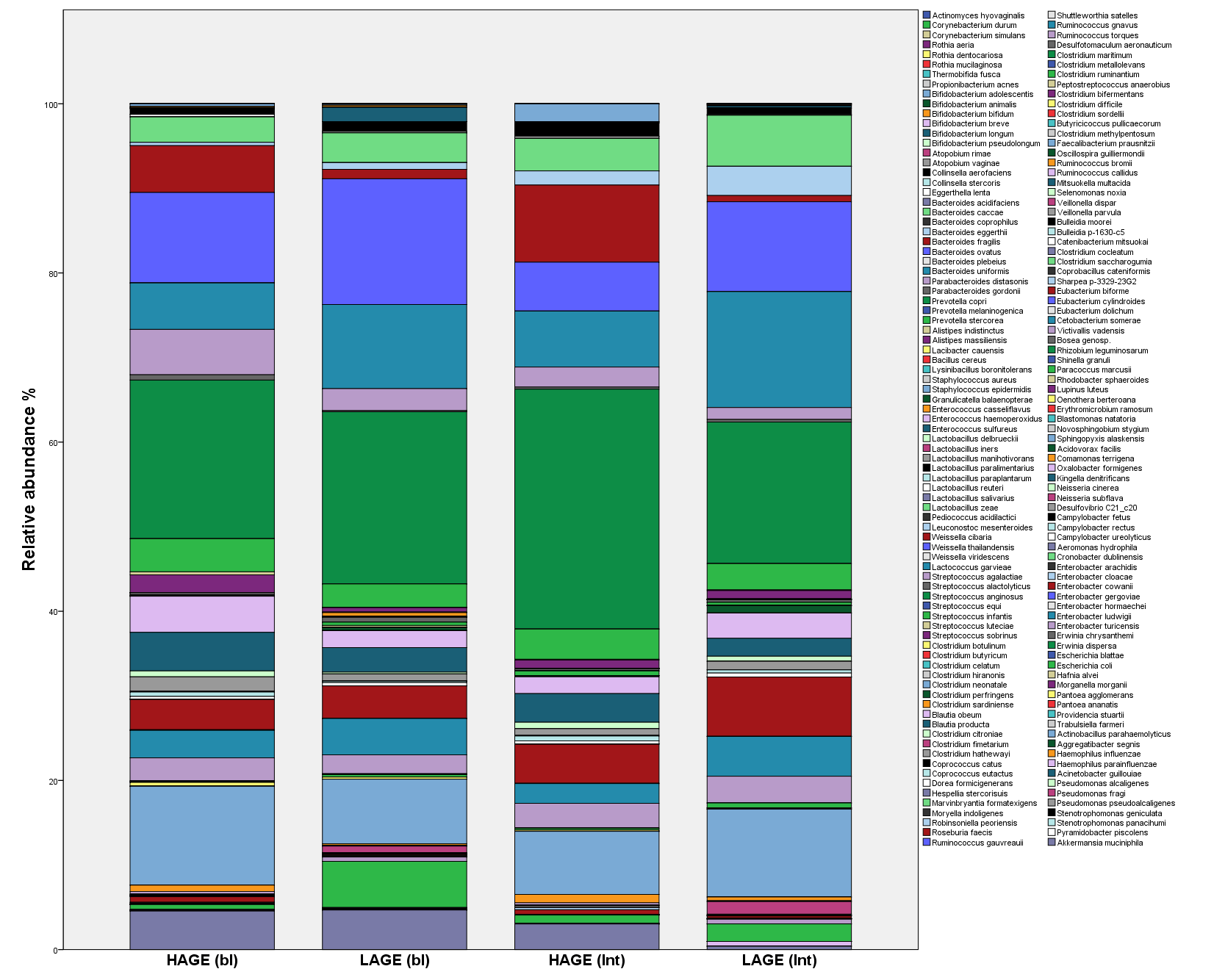

Supplement: S2 Fig — (TIF) [file pone.0184789.s002.tif]
