# Supplementary material for: Advanced glycation end products dietary restriction effects on bacterial gut microbiota in peritoneal dialysis patients; a randomized open label controlled trial
Source: PLoS One. 2017 Sep 20;12(9):e0184789. doi: 10.1371/journal.pone.0184789 (PMC5607175; doi:10.1371/journal.pone.0184789)
Supplement: S1 Table — (DOCX) [file pone.0184789.s003.docx]

S1 Table. Relative abundance of bacterial phyla at baseline

| Phyla | Mean relative abundance % | SD |
| --- | --- | --- |
| Bacteroidetes | 56.47474 | 18.53783 |
| Firmicutes | 32.725 | 13.53911 |
| Verrucomicrobia | 4.620555 | 10.23666 |
| Proteobacteria | 3.660232 | 8.493787 |
| Actinobacteria | 2.50807 | 4.171324 |
| Synergistetes | 0.010645 | 0.044112 |
| Lentisphaerae | 0.000755 | 0.002258 |
| Fusobacteria | 0 | 0 |
