# Supplementary material for: Advanced glycation end products dietary restriction effects on bacterial gut microbiota in peritoneal dialysis patients; a randomized open label controlled trial
Source: PLoS One. 2017 Sep 20;12(9):e0184789. doi: 10.1371/journal.pone.0184789 (PMC5607175; doi:10.1371/journal.pone.0184789)
Supplement: S2 Table — (DOCX) [file pone.0184789.s004.docx]

S2 Table. Relative abundance of bacterial genus at baseline

| Phyla | Mean relative abundance % | SD |
| --- | --- | --- |
| Bacteroides | 27.76647 | 23.32351 |
| Prevotella | 22.89615 | 29.14888 |
| Faecalibacterium | 9.610344 | 9.487609 |
| Blautia | 6.800082 | 5.195223 |
| Ruminococcus | 6.272934 | 4.371949 |
| Akkermansia | 4.620555 | 10.23666 |
| Parabacteroides | 4.314679 | 8.406122 |
| Roseburia | 3.709788 | 4.340508 |
| Escherichia | 3.112766 | 8.363284 |
| Clostridium | 2.306715 | 1.75105 |
| Alistipes | 1.497327 | 4.387789 |
| Bifidobacterium | 1.348835 | 3.717721 |
| Collinsella | 0.93716 | 1.834474 |
| Streptococcus | 0.791403 | 1.751961 |
| Ruminococcus | 0.696372 | 1.370398 |
| Enterococcus | 0.530453 | 1.541365 |
| Eubacterium | 0.519198 | 1.00806 |
| Veillonella | 0.469762 | 1.039801 |
| Comamonas | 0.411639 | 0.835319 |
| Clostridium | 0.344064 | 0.597323 |
| Dorea | 0.340858 | 0.44434 |
| Eggerthella | 0.213066 | 0.33106 |
| Haemophilus | 0.115518 | 0.275786 |
| Morganella | 0.082431 | 0.384094 |
| Sharpea | 0.051717 | 0.168419 |
| Weissella | 0.030901 | 0.165601 |
| Catenibacterium | 0.026281 | 0.081912 |
| Campylobacter | 0.02267 | 0.115695 |
| Robinsoniella | 0.022386 | 0.075375 |
| Granulicatella | 0.022236 | 0.045662 |
| Mitsuokella | 0.014379 | 0.05937 |
| Oxalobacter | 0.011297 | 0.024212 |
| Lactobacillus | 0.010926 | 0.036447 |
| Pyramidobacter | 0.010645 | 0.044112 |
| Marvinbryantia | 0.010009 | 0.016071 |
| Acidovorax | 0.008896 | 0.048027 |
| Trabulsiella | 0.007566 | 0.035744 |
| Staphylococcus | 0.006032 | 0.01895 |
| Rothia | 0.004203 | 0.011236 |
| Butyricicoccus | 0.004066 | 0.011879 |
| Bulleidia | 0.003754 | 0.016941 |
| Atopobium | 0.003083 | 0.012794 |
| Pseudomonas | 0.002844 | 0.013783 |
| Shinella | 0.002705 | 0.016005 |
| Aggregatibacter | 0.002349 | 0.007123 |
| Peptostreptococcus | 0.001278 | 0.002093 |
| Leuconostoc | 0.001184 | 0.005774 |
| Corynebacterium | 0.001086 | 0.002082 |
| Lactococcus | 0.000963 | 0.003797 |
| Victivallis | 0.000755 | 0.002258 |
| Hespellia | 0.000702 | 0.00201 |
| Actinomyces | 0.000637 | 0.001782 |
| Rhodobacter | 0.000624 | 0.001946 |
| Erwinia | 0.000623 | 0.002681 |
| Neisseria | 0.000622 | 0.00172 |
| Providencia | 0.000485 | 0.002356 |
| Hafnia | 0.000451 | 0.001882 |
| Bosea | 0.000386 | 0.002286 |
| Bacillus | 0.000376 | 0.00135 |
| Blastomonas | 0.000326 | 0.001197 |
| Pediococcus | 0.000269 | 0.00111 |
| Kingella | 0.000264 | 0.0008 |
| Desulfotomaculum | 0.000243 | 0.001011 |
| Cronobacter | 0.000177 | 0.00073 |
| Pantoea | 0.000173 | 0.001024 |
| Sphingopyxis | 0.000155 | 0.000914 |
| Oscillospira | 0.000137 | 0.000813 |
| Lacibacter | 0.000114 | 0.000677 |
| Stenotrophomonas | 0.000113 | 0.000471 |
| Shuttleworthia | 0.000106 | 0.00044 |
| Desulfovibrio | 6.56E-05 | 0.000388 |
| Oenothera | 6.21E-05 | 0.000367 |
| Lupinus | 6.04E-05 | 0.000358 |
| Novosphingobium | 4.82E-05 | 0.000285 |
| Thermobifida | 0 | 0 |
| Selenomonas | 0 | 0 |
| Rhizobium | 0 | 0 |
| Propionibacterium | 0 | 0 |
| Paracoccus | 0 | 0 |
| Moryella | 0 | 0 |
| Lysinibacillus | 0 | 0 |
| Erythromicrobium | 0 | 0 |
| Cetobacterium | 0 | 0 |
| Aeromonas | 0 | 0 |
| Actinobacillus | 0 | 0 |
| Acinetobacter | 0 | 0 |
