# Supplementary material for: Advanced glycation end products dietary restriction effects on bacterial gut microbiota in peritoneal dialysis patients; a randomized open label controlled trial
Source: PLoS One. 2017 Sep 20;12(9):e0184789. doi: 10.1371/journal.pone.0184789 (PMC5607175; doi:10.1371/journal.pone.0184789)
Supplement: S3 Table — (DOCX) [file pone.0184789.s005.docx]

S3 Table. Relative abundance of bacterial species at baseline

| Bacterial Species | Mean relative abundance % | SD |
| --- | --- | --- |
| Prevotella copri | 23.08629911 | 27.48688 |
| Faecalibacterium prausnitzii | 9.781570515 | 9.151897 |
| Bacteroides ovatus | 8.468244036 | 16.8459 |
| Bacteroides fragilis | 7.158073197 | 13.86008 |
| Bacteroides uniformis | 6.005629579 | 8.106592 |
| Roseburia faecis | 4.038153998 | 3.505482 |
| Blautia producta | 4.030358288 | 3.680627 |
| Parabacteroides distasonis | 4.006236023 | 8.753994 |
| Akkermansia muciniphila | 3.870253604 | 6.701038 |
| Prevotella stercorea | 3.765915019 | 6.569003 |
| Bacteroides caccae | 3.383650853 | 6.522095 |
| Blautia obeum | 3.236473044 | 3.1231 |
| Ruminococcus gnavus | 2.839613625 | 2.276818 |
| Ruminococcus torques | 2.823694234 | 3.089096 |
| Alistipes massiliensis | 1.584692648 | 4.406808 |
| Clostridium hathewayi | 1.278003327 | 1.412622 |
| Bifidobacterium adolescentis | 1.123618057 | 3.472104 |
| Collinsella aerofaciens | 1.082557832 | 3.29091 |
| Bacteroides eggerthii | 0.97131585 | 3.975982 |
| Ruminococcus bromii | 0.870643991 | 2.226521 |
| Escherichia coli | 0.771820703 | 1.626173 |
| Clostridium citroniae | 0.734604613 | 1.350319 |
| Eubacterium biforme | 0.591747677 | 1.166337 |
| Coprococcus eutactus | 0.541666055 | 1.059616 |
| Parabacteroides gordonii | 0.458898836 | 1.308719 |
| Dorea formicigenerans | 0.326310463 | 0.413524 |
| Streptococcus infantis | 0.319662008 | 1.26341 |
| Clostridium difficile | 0.312440837 | 0.500853 |
| Ruminococcus callidus | 0.269072475 | 0.386146 |
| Eggerthella lenta | 0.250567746 | 0.401704 |
| Alistipes indistinctus | 0.240234119 | 0.520295 |
| Streptococcus alactolyticus | 0.229395999 | 0.455016 |
| Clostridium ruminantium | 0.170715556 | 0.260966 |
| Sharpea p-3329-23G2 | 0.134839224 | 0.576584 |
| Catenibacterium mitsuokai | 0.116635178 | 0.287298 |
| Bifidobacterium bifidum | 0.108497539 | 0.342656 |
| Haemophilus parainfluenzae | 0.106189013 | 0.256724 |
| Coprococcus catus | 0.096384836 | 0.138726 |
| Eubacterium dolichum | 0.095856954 | 0.206275 |
| Clostridium perfringens | 0.091940674 | 0.181679 |
| Veillonella dispar | 0.079849119 | 0.111542 |
| Bifidobacterium longum | 0.07676224 | 0.165288 |
| Ruminococcus gauvreauii | 0.061433257 | 0.101907 |
| Clostridium saccharogumia | 0.050684197 | 0.09314 |
| Eubacterium cylindroides | 0.03766432 | 0.122109 |
| Robinsoniella peoriensis | 0.036632668 | 0.094546 |
| Campylobacter ureolyticus | 0.024884692 | 0.12298 |
| Granulicatella balaenopterae | 0.024346545 | 0.087317 |
| Atopobium rimae | 0.022828126 | 0.120831 |
| Clostridium metallolevans | 0.019304829 | 0.031536 |
| Bacteroides acidifaciens | 0.0177828 | 0.029733 |
| Oxalobacter formigenes | 0.017695915 | 0.026779 |
| Mitsuokella multacida | 0.016416954 | 0.062918 |
| Veillonella parvula | 0.016294988 | 0.028642 |
| Marvinbryantia formatexigens | 0.014373459 | 0.01709 |
| Streptococcus anginosus | 0.014130049 | 0.060591 |
| Pyramidobacter piscolens | 0.012907371 | 0.046815 |
| Clostridium cocleatum | 0.008642627 | 0.020217 |
| Enterobacter cloacae | 0.006776965 | 0.017911 |
| Bulleidia p-1630-c5 | 0.006106168 | 0.020289 |
| Collinsella stercoris | 0.005251793 | 0.026641 |
| Enterococcus casseliflavus | 0.004767844 | 0.010041 |
| Enterobacter turicensis | 0.004451528 | 0.011186 |
| Butyricicoccus pullicaecorum | 0.004032957 | 0.011001 |
| Rothia mucilaginosa | 0.003112992 | 0.007573 |
| Aggregatibacter segnis | 0.002799553 | 0.007595 |
| Leuconostoc mesenteroides | 0.002623125 | 0.007559 |
| Bifidobacterium animalis | 0.002588671 | 0.006406 |
| Neisseria subflava | 0.002291234 | 0.006971 |
| Coprobacillus cateniformis | 0.002245875 | 0.00606 |
| Bifidobacterium breve | 0.001986361 | 0.007636 |
| Actinomyces hyovaginalis | 0.001928799 | 0.008234 |
| Lactobacillus zeae | 0.001735583 | 0.003565 |
| Enterobacter ludwigii | 0.001585113 | 0.007265 |
| Streptococcus luteciae | 0.001580044 | 0.003328 |
| Clostridium methylpentosum | 0.001411023 | 0.003401 |
| Campylobacter fetus | 0.001314428 | 0.004676 |
| Enterobacter gergoviae | 0.001069797 | 0.005228 |
| Lactococcus garvieae | 0.000853896 | 0.003957 |
| Corynebacterium durum | 0.000829296 | 0.001548 |
| Victivallis vadensis | 0.000777098 | 0.002733 |
| Staphylococcus epidermidis | 0.000766305 | 0.003019 |
| Rothia aeria | 0.00072941 | 0.002568 |
| Peptostreptococcus anaerobius | 0.000726164 | 0.001692 |
| Hespellia stercorisuis | 0.000649458 | 0.001651 |
| Lactobacillus paraplantarum | 0.000619231 | 0.001687 |
| Bulleidia moorei | 0.00057668 | 0.001379 |
| Streptococcus sobrinus | 0.000481905 | 0.002299 |
| Oscillospira guilliermondii | 0.000474962 | 0.001699 |
| Streptococcus agalactiae | 0.000461965 | 0.002087 |
| Lactobacillus manihotivorans | 0.000458495 | 0.001798 |
| Comamonas terrigena | 0.00042739 | 0.001589 |
| Pseudomonas alcaligenes | 0.000363857 | 0.001194 |
| Clostridium sordellii | 0.0003296 | 0.000871 |
| Blastomonas natatoria | 0.000298808 | 0.001112 |
| Clostridium bifermentans | 0.000290575 | 0.000777 |
| Pediococcus acidilactici | 0.00028056 | 0.001131 |
| Pseudomonas fragi | 0.000265171 | 0.001476 |
| Clostridium maritimum | 0.00026196 | 0.001073 |
| Morganella morganii | 0.000248095 | 0.000814 |
| Bacillus cereus | 0.000244756 | 0.000909 |
| Kingella denitrificans | 0.000244016 | 0.000806 |
| Cetobacterium somerae | 0.000237314 | 0.001321 |
| Campylobacter rectus | 0.000220937 | 0.001086 |
| Rhodobacter sphaeroides | 0.000213706 | 0.0009 |
| Weissella cibaria | 0.000207343 | 0.000821 |
| Streptococcus equi | 0.000182651 | 0.001017 |
| Bacteroides coprophilus | 0.000180645 | 0.000627 |
| Bacteroides plebeius | 0.000174708 | 0.000973 |
| Clostridium sardiniense | 0.000155492 | 0.000607 |
| Desulfotomaculum aeronauticum | 0.000155064 | 0.000863 |
| Aeromonas hydrophila | 0.000151113 | 0.000841 |
| Clostridium neonatale | 0.000139418 | 0.000549 |
| Lactobacillus reuteri | 0.0001372 | 0.000533 |
| Clostridium botulinum | 0.000133556 | 0.000518 |
| Haemophilus influenzae | 0.000129397 | 0.00072 |
| Shuttleworthia satelles | 0.000119331 | 0.000467 |
| Cronobacter dublinensis | 0.000102184 | 0.000569 |
| Prevotella melaninogenica | 0.000100753 | 0.000561 |
| Lactobacillus paralimentarius | 9.66621E-05 | 0.000538 |
| Acinetobacter guillouiae | 8.83904E-05 | 0.000492 |
| Selenomonas noxia | 8.57768E-05 | 0.000345 |
| Lactobacillus iners | 8.21568E-05 | 0.000457 |
| Desulfovibrio C21_c20 | 7.41207E-05 | 0.000413 |
| Stenotrophomonas panacihumi | 7.31674E-05 | 0.000407 |
| Lactobacillus delbrueckii | 7.31674E-05 | 0.000407 |
| Lupinus luteus | 6.82393E-05 | 0.00038 |
| Enterococcus haemoperoxidus | 6.46986E-05 | 0.00036 |
| Neisseria cinerea | 5.10922E-05 | 0.000284 |
| Clostridium hiranonis | 4.38748E-05 | 0.000244 |
| Escherichia blattae | 2.68412E-05 | 0.000149 |
