# Supplementary material for: Advanced glycation end products dietary restriction effects on bacterial gut microbiota in peritoneal dialysis patients; a randomized open label controlled trial
Source: PLoS One. 2017 Sep 20;12(9):e0184789. doi: 10.1371/journal.pone.0184789 (PMC5607175; doi:10.1371/journal.pone.0184789)
Supplement: S4 Table — (DOCX) [file pone.0184789.s006.docx]

S4 Table: Bacterial Species Principle Components Coefficients

| Bacterial Species | Bacterial Species Principle Components Coefficients | | |
| --- | --- | --- | --- |
|  | PCA1 | PCA2 | PCA3 |
| s__luteciae | 0.952825 |  |  |
| s__cibaria | 0.950993 |  |  |
| s__casseliflavus | 0.923926 |  |  |
| s__mesenteroides | 0.922616 |  |  |
| s__parainfluenzae | 0.920215 |  |  |
| s__gnavus | 0.918032 |  |  |
| s__alactolyticus | 0.871602 |  |  |
| s__agglomerans | 0.854506 |  |  |
| s__ananatis | 0.845408 |  |  |
| s__dispersa | 0.845408 |  |  |
| s__cinerea | 0.845408 |  |  |
| s__butyricum | 0.845408 |  |  |
| s__fimetarium | 0.845408 |  |  |
| s__dentocariosa | 0.817589 |  |  |
| s__bifidum | 0.776184 |  |  |
| s__durum | 0.734441 |  |  |
| s__obeum | 0.704061 |  |  |
| s__pullicaecorum | 0.700108 |  |  |
| s__morganii | 0.661069 |  |  |
| s__faecis | 0.630959 |  |  |
| s__mucilaginosa | 0.539918 |  | -0.38403 |
| s__longum | 0.520077 |  |  |
| s__cloacae | 0.48945 | 0.31237 |  |
| s__denitrificans | 0.475163 |  |  |
| s__prausnitzii | -0.37006 |  |  |
| s__rimae |  | 0.971004 |  |
| s__hyovaginalis |  | 0.961437 |  |
| s__p-3329-23G2 |  | 0.95203 |  |
| s__anginosus |  | 0.949608 |  |
| s__aeria |  | 0.946186 |  |
| s__cylindroides |  | 0.946186 |  |
| s__agalactiae |  | 0.946186 |  |
| s__multacida |  | 0.946186 |  |
| s__gauvreauii |  | 0.936589 |  |
| s__aerofaciens |  | 0.934619 |  |
| s__infantis |  | 0.930717 |  |
| s__balaenopterae |  | 0.907795 |  |
| s__biforme |  | 0.906626 |  |
| s__mitsuokai |  | 0.80613 | -0.41214 |
| s__equi |  | 0.798263 |  |
| s__difficile |  | 0.640695 |  |
| s__formatexigens |  | 0.542348 | -0.52125 |
| s__formicigenerans |  | 0.41538 |  |
| s__torques |  | 0.392751 |  |
| s__stercorea |  |  | -0.70203 |
| s__saccharogumia | 0.435606 |  | 0.668527 |
| s__reuteri |  |  | 0.661372 |
| s__p-1630-c5 |  |  | -0.65258 |
| s__peoriensis |  |  | -0.65059 |
| s__ovatus |  |  | 0.627818 |
| s__cocleatum |  |  | 0.603912 |
| s__subflava |  |  | -0.60325 |
| s__catus |  |  | -0.6025 |
| s__sardiniense |  |  | 0.595831 |
| s__coprophilus |  |  | 0.584919 |
| s__copri |  |  | -0.58026 |
| s__satelles |  |  | 0.569416 |
| s__moorei |  |  | 0.567302 |
| s__parvula |  |  | 0.567169 |
| s__callidus |  |  | -0.53342 |
| s__anaerobius |  |  | 0.531597 |
| s__hydrophila |  |  | -0.51935 |
| s__sphaeroides |  |  | -0.51935 |
| s__cereus |  |  | -0.51102 |
| s__zeae |  |  | 0.505869 |
| s__perfringens |  |  | 0.50028 |
| s__segnis | 0.486932 |  | -0.48971 |
| s__acidifaciens |  |  | 0.45702 |
| s__fetus |  |  | -0.43759 |
| s__citroniae |  |  | 0.404483 |
| s__eggerthii |  |  | 0.389663 |
| s__uniformis |  |  | 0.379727 |
| s__formigenes |  |  | -0.36548 |
| s__eutactus |  |  | -0.34352 |
| s__hathewayi |  |  | -0.34277 |
| s__noxia |  |  | -0.34175 |
| s__dolichum |  |  | 0.334431 |
