# Supplementary material for: Advanced glycation end products dietary restriction effects on bacterial gut microbiota in peritoneal dialysis patients; a randomized open label controlled trial
Source: PLoS One. 2017 Sep 20;12(9):e0184789. doi: 10.1371/journal.pone.0184789 (PMC5607175; doi:10.1371/journal.pone.0184789)
Supplement: S5 Table — (PDF) [file pone.0184789.s007.pdf]

	[illegible]	[illegible]	s__aeria	[illegible]	[illegible]	s__acnes	[illegible]	[illegible]	[illegible]	s__breve	[illegible]
s__hyovaginalis	1	-0.05	0.982	0.047	-0.002	-0.045	-0.034	0.143	-0.019	-0.045	[illegible]
s__durum	-0.05	1	-0.077	0.366	0.442	-0.077	-0.084	-0.122	0.696	-0.077	[illegible]
s__aeria	0.982	-0.077	1	-0.043	-0.087	-0.036	-0.022	0.159	-0.064	-0.036	[illegible]
s__dentocariosa	0.047	0.366	-0.043	1	0.455	-0.043	-0.056	-0.068	0.539	-0.043	[illegible]
s__mucilaginosa	-0.002	0.442	-0.087	0.455	1	-0.087	-0.071	-0.101	0.284	-0.087	[illegible]
s__acnes	-0.045	-0.077	-0.036	-0.043	-0.087	1	-0.041	-0.057	-0.064	-0.036	[illegible]
s__adolescentis	-0.034	-0.084	-0.022	-0.056	-0.071	-0.041	1	0.96	-0.02	0.126	[illegible]
s__animalis	0.143	-0.122	0.159	-0.068	-0.101	-0.057	0.96	1	-0.102	0.22	[illegible]
s__bifidum	-0.019	0.696	-0.064	0.539	0.284	-0.064	-0.02	-0.102	1	-0.062	[illegible]
s__breve	-0.045	-0.077	-0.036	-0.043	-0.087	-0.036	0.126	0.22	-0.062	1	[illegible]
s__longum	0.163	0.311	0.137	0.39	0.119	-0.069	0.059	0.145	0.452	0.786	[illegible]
s__rimae	0.995	-0.089	0.987	-0.049	-0.048	-0.043	-0.031	0.147	-0.077	-0.042	[illegible]
s__aerofaciens	0.938	-0.088	0.956	-0.056	-0.041	-0.061	-0.065	0.104	-0.124	-0.069	[illegible]
s__lenta	-0.112	0.006	-0.103	0.05	-0.092	0.051	0.888	0.849	0.096	0.167	[illegible]
s__acidifaciens	-0.024	-0.211	-0.102	-0.122	-0.044	-0.056	-0.132	-0.117	-0.184	-0.102	[illegible]
s__caccae	-0.112	-0.014	-0.102	0.04	-0.132	0.517	-0.127	-0.124	0.011	-0.103	[illegible]
s__coprophilus	0.1	-0.103	-0.048	-0.058	0.179	-0.048	-0.061	-0.076	-0.087	-0.048	[illegible]
s__eggerthii	-0.002	-0.139	-0.064	-0.076	-0.015	-0.066	-0.087	-0.105	-0.121	-0.065	[illegible]
s__fragilis	-0.085	-0.051	-0.068	-0.083	-0.138	-0.067	0.749	0.627	0.302	-0.069	[illegible]
s__ovatus	-0.054	0.092	-0.145	0.13	0.043	0.308	-0.202	-0.169	0.117	-0.159	[illegible]
s__uniformis	-0.106	-0.148	-0.095	-0.071	-0.211	-0.004	0.004	0.107	-0.121	0.844	[illegible]
s__distasonis	-0.046	-0.059	-0.06	-0.105	-0.154	0.048	-0.023	-0.128	0.409	-0.097	[illegible]
s__gordonii	-0.075	-0.165	-0.057	0.005	-0.181	-0.077	-0.102	-0.12	-0.141	-0.078	[illegible]
s__copri	-0.036	-0.11	0.006	-0.139	0.038	-0.156	-0.208	-0.226	-0.295	-0.165	[illegible]
s__melaninogenica	-0.045	-0.077	-0.036	-0.043	-0.087	-0.036	-0.046	-0.057	-0.064	-0.036	[illegible]
s__stercorea	-0.117	0.017	-0.095	-0.061	0.514	-0.11	-0.146	-0.179	-0.206	-0.115	[illegible]
s__indistinctus	-0.098	-0.202	-0.074	-0.091	-0.224	0.152	-0.121	-0.095	-0.17	-0.094	[illegible]
s__massiliensis	-0.113	-0.101	-0.093	-0.051	-0.177	0.135	-0.123	-0.104	-0.094	-0.096	[illegible]
s__cereus	-0.053	0.384	-0.042	-0.051	0.381	-0.042	-0.053	-0.067	-0.077	-0.042	[illegible]
s__epidermidis	0.018	0.328	-0.049	-0.059	0.468	-0.049	-0.061	-0.078	-0.088	-0.049	[illegible]
s__balaenopterae	0.906	0.068	0.832	0.027	0.111	-0.069	-0.065	0.083	0.062	-0.067	[illegible]
s__casseliflavus	0.011	0.808	-0.043	0.604	0.369	-0.055	-0.056	-0.069	0.801	-0.053	[illegible]
s__manihotivorans	-0.045	-0.077	-0.036	-0.043	-0.087	-0.036	-0.047	-0.057	-0.064	-0.036	[illegible]
s__paraplantarum	-0.05	-0.029	-0.04	-0.048	-0.085	0.997	-0.046	-0.063	-0.027	-0.04	[illegible]
s__reuteri	0.026	-0.158	-0.074	-0.089	0.04	0.607	-0.066	-0.074	-0.133	0.086	[illegible]
s__salivarius	-0.077	-0.132	-0.061	0.009	-0.145	-0.061	-0.08	-0.097	-0.111	-0.061	[illegible]
s__zeae	0.025	-0.042	-0.092	-0.029	0.164	0.409	-0.082	-0.106	0.027	0.055	[illegible]
s__acidilactici	-0.077	-0.132	-0.061	-0.074	-0.103	-0.061	0.752	0.703	-0.111	-0.061	[illegible]
s__mesenteroides	0.267	0.749	0.214	0.632	0.355	-0.063	-0.076	-0.035	0.768	-0.063	[illegible]
s__cibaria	0.044	0.57	-0.044	0.937	0.488	-0.044	-0.058	-0.071	0.698	-0.044	[illegible]
s__agalactiae	0.982	-0.077	1	-0.043	-0.087	-0.036	-0.022	0.159	-0.064	-0.036	[illegible]
s__alactolyticus	0.058	0.802	-0.005	0.675	0.748	-0.081	-0.1	-0.109	0.614	-0.059	[illegible]
s__anginosus	0.984	0.063	0.981	0.039	0	-0.052	-0.043	0.132	0.066	-0.052	[illegible]
s__equi	0.804	0.334	0.824	-0.061	-0.033	-0.05	-0.045	0.102	0.262	-0.05	[illegible]
s__infantis	0.954	0.121	0.894	0.141	0.135	-0.063	-0.062	0.097	0.13	-0.065	[illegible]
s__luteciae	0.108	0.816	0.05	0.701	0.494	-0.07	-0.088	-0.086	0.776	-0.07	[illegible]
s__botulinum	-0.045	-0.077	-0.036	-0.043	-0.087	-0.036	0.126	0.22	-0.062	1	[illegible]
s__butyricum	0.058	0.389	-0.036	0.976	0.482	-0.036	-0.047	-0.057	0.562	-0.036	[illegible]
s__celatum	-0.045	-0.077	-0.036	-0.043	-0.054	-0.036	-0.047	-0.057	-0.064	-0.036	[illegible]
s__hiranonis	-0.052	-0.065	-0.041	-0.05	-0.101	-0.041	-0.036	-0.066	0.028	-0.041	[illegible]
s__perfringens	0.074	-0.106	-0.077	-0.018	0.178	-0.077	-0.098	-0.116	-0.075	-0.074	[illegible]
s__sardiniense	0.067	-0.13	-0.061	-0.073	0.117	-0.061	-0.045	-0.045	-0.109	0.132	[illegible]
s__obeum	0.217	0.561	0.179	0.676	0.505	0.288	-0.214	-0.158	0.428	-0.196	[illegible]
s__producta	0.049	0.124	0.023	0.186	0.172	-0.077	0.877	0.809	0.195	-0.031	[illegible]
s__citroniae	-0.137	-0.158	-0.117	-0.083	-0.23	0.763	0.055	0.11	-0.097	0.091	[illegible]
s__fimetarium	0.058	0.389	-0.036	0.976	0.482	-0.036	-0.047	-0.057	0.562	-0.036	[illegible]
s__hathewayi	-0.155	0.084	-0.119	-0.11	-0.06	-0.128	-0.107	-0.147	-0.207	-0.126	[illegible]
s__catus	0.106	0.122	0.097	-0.104	0.697	-0.089	-0.122	-0.119	-0.181	-0.101	[illegible]
s__eutactus	-0.099	0.197	-0.079	-0.095	0.073	-0.075	-0.101	-0.126	-0.131	-0.079	[illegible]
s__formicigenerans	0.381	0.053	0.394	-0.113	-0.07	-0.053	0.083	0.131	-0.245	-0.141	[illegible]
s__stercorisuis	0.274	0.154	0.296	-0.002	-0.108	-0.072	-0.085	0.006	-0.13	-0.072	[illegible]
s__formatexigens	0.429	-0.104	0.465	-0.071	0.254	-0.124	-0.145	-0.074	-0.223	-0.124	[illegible]
s__peoriensis	-0.078	0.367	-0.066	-0.009	0.653	-0.066	0	-0.013	-0.055	0.059	[illegible]
s__faecis	-0.166	0.639	-0.174	0.381	0.167	-0.175	-0.185	-0.202	0.64	-0.186	[illegible]
s__gauvreauii	0.9	-0.001	0.92	-0.058	0.133	-0.088	-0.055	0.101	-0.111	-0.085	[illegible]
s__satelles	0.123	-0.077	-0.036	-0.043	0.223	-0.036	-0.047	-0.057	-0.064	-0.036	[illegible]
s__gnavus	0.071	0.71	-0.014	0.68	0.473	-0.058	0.033	0.024	0.696	-0.042	[illegible]
s__torques	0.366	-0.202	0.391	-0.08	-0.105	0.102	-0.057	0.026	-0.222	-0.124	[illegible]
s__aeronauticum	-0.072	-0.124	-0.058	-0.012	-0.14	0.837	-0.07	-0.091	-0.104	-0.058	[illegible]
s__metallolevans	0.142	-0.121	0.166	-0.103	-0.181	-0.086	-0.039	0.022	-0.103	0.31	[illegible]
s__ruminantium	0	0.101	-0.115	0.17	0.284	-0.115	-0.138	-0.169	0.102	-0.096	[illegible]
s__anaerobius	0.104	-0.023	-0.049	-0.059	0.206	-0.049	-0.055	-0.063	-0.088	0.007	[illegible]
s__bifermentans	-0.045	-0.077	-0.036	-0.043	-0.085	-0.036	-0.042	-0.057	-0.065	-0.036	[illegible]
s__difficile	0.651	-0.159	0.662	-0.092	-0.16	-0.097	0.097	0.272	-0.025	0.536	[illegible]
s__sordellii	-0.048	0.285	-0.074	0.36	0.16	-0.074	-0.097	-0.118	0.142	-0.074	[illegible]
s__pullicaecorum	0.004	0.348	-0.067	0.791	0.35	-0.067	0.093	0.067	0.49	-0.067	[illegible]
s__methylpentosum	-0.08	-0.1	-0.064	-0.076	-0.148	0.025	-0.082	-0.101	-0.086	-0.064	[illegible]
s__prausnitzii	-0.081	-0.204	-0.076	-0.203	-0.21	-0.076	-0.208	-0.303	-0.044	-0.208	[illegible]
s__guilliermondii	-0.055	-0.095	-0.044	-0.053	-0.076	-0.044	-0.058	-0.07	-0.08	-0.044	[illegible]
s__bromii	-0.055	-0.122	-0.038	-0.081	-0.156	-0.064	-0.083	0.058	-0.1	-0.067	[illegible]
s__callidus	-0.114	0.284	-0.091	-0.109	0.443	-0.084	-0.076	-0.114	0.066	-0.091	[illegible]
s__multacida	0.982	-0.077	1	-0.043	-0.087	-0.036	-0.022	0.159	-0.064	-0.036	[illegible]
s__noxia	-0.077	-0.132	-0.062	0.093	0.318	-0.062	-0.078	-0.098	-0.11	-0.062	[illegible]
s__dispar	-0.066	-0.103	-0.06	-0.061	-0.109	-0.059	-0.061	-0.068	-0.09	0.04	[illegible]
s__parvula	0.081	-0.116	-0.066	-0.08	0.207	-0.066	-0.054	-0.053	-0.119	0.126	[illegible]
s__moorei	0.107	-0.1	-0.046	-0.056	0.199	-0.046	-0.042	-0.044	-0.084	0.065	[illegible]
s__p-1630-c5	-0.059	0.348	-0.047	-0.057	0.637	-0.047	-0.058	-0.075	-0.085	-0.047	[illegible]
s__mitsuokai	0.714	0.03	0.732	-0.075	0.474	-0.062	-0.059	0.066	-0.112	-0.062	[illegible]
s__cocleatum	0.098	-0.008	-0.059	-0.025	0.213	-0.059	-0.024	-0.044	0.002	-0.054	[illegible]
s__saccharogumia	0.308	0.273	0.197	0.258	0.19	0.326	0.008	0.095	0.304	0.375	[illegible]
s__cateniformis	-0.075	-0.128	-0.06	0.137	-0.134	0.305	0.223	0.204	-0.107	-0.025	[illegible]
s__p-3329-23G2	0.978	-0.067	0.997	-0.049	-0.053	-0.045	-0.034	0.145	-0.082	-0.045	[illegible]
s__biforme	0.811	-0.053	0.782	-0.077	0.393	-0.088	-0.092	0.04	-0.159	-0.088	[illegible]
s__cylindroides	0.982	-0.077	1	-0.043	-0.087	-0.036	-0.022	0.159	-0.064	-0.036	[illegible]
s__dolichum	-0.005	0.038	0.001	0.045	-0.133	0.028	0.187	0.218	0.073	0.453	[illegible]
s__vadensis	-0.087	-0.15	-0.07	-0.084	-0.165	-0.07	-0.091	-0.111	-0.122	-0.07	[illegible]
s__leguminosarum	-0.045	-0.077	-0.036	-0.043	-0.087	1	-0.041	-0.057	-0.064	-0.036	[illegible]
s__sphaeroides	-0.045	0.405	-0.036	-0.043	0.404	-0.036	-0.044	-0.057	-0.064	-0.036	[illegible]
s__natatoria	-0.045	-0.077	-0.036	-0.043	-0.048	-0.036	0.981	0.933	-0.064	-0.036	[illegible]
s__terrigena	-0.056	-0.096	-0.045	-0.054	-0.061	-0.045	-0.058	-0.071	-0.081	-0.045	[illegible]
s__formigenes	-0.042	-0.003	-0.019	-0.106	0.441	-0.089	-0.111	-0.11	-0.159	-0.089	[illegible]
s__denitrificans	-0.045	0.705	-0.036	-0.043	0.072	-0.036	-0.046	-0.057	0.558	-0.036	[illegible]
s__cinerea	0.058	0.389	-0.036	0.976	0.482	-0.036	-0.047	-0.057	0.562	-0.036	[illegible]
s__subflava	-0.081	0.173	-0.065	-0.078	0.758	-0.065	-0.08	-0.103	-0.055	-0.065	[illegible]
s__fetus	-0.045	-0.052	-0.043	0.038	0.697	-0.043	-0.053	-0.069	-0.022	-0.043	[illegible]
s__rectus	-0.045	-0.077	-0.036	-0.043	-0.052	-0.036	-0.047	-0.057	-0.064	-0.036	[illegible]
s__hydrophila	-0.045	0.405	-0.036	-0.043	0.404	-0.036	-0.044	-0.057	-0.064	-0.036	[illegible]
s__dublinensis	-0.053	-0.09	-0.042	-0.05	-0.102	-0.042	-0.055	-0.067	-0.076	-0.042	[illegible]
s__cloacae	0.317	0.317	0.307	0.274	0.235	0.032	-0.125	-0.081	0.297	-0.105	[illegible]
s__gergoviae	-0.073	-0.126	-0.059	-0.07	-0.132	-0.059	0.352	0.413	-0.104	0.812	[illegible]
s__ludwigii	0.047	-0.062	0.058	-0.019	-0.108	-0.06	-0.075	-0.071	-0.042	-0.06	[illegible]
s__turicensis	-0.048	-0.069	-0.04	-0.037	-0.077	0.999	-0.046	-0.063	-0.059	-0.04	[illegible]
s__chrysanthemi	-0.045	-0.077	-0.036	-0.043	-0.087	-0.036	-0.046	-0.057	-0.064	-0.036	[illegible]
s__dispersa	0.058	0.389	-0.036	0.976	0.482	-0.036	-0.047	-0.057	0.562	-0.036	[illegible]
s__blattae	-0.045	-0.077	-0.036	-0.043	-0.052	-0.036	-0.047	-0.057	-0.064	-0.036	[illegible]
s__coli	-0.008	-0.073	0.003	-0.023	-0.12	-0.073	0.119	0.105	-0.049	-0.058	[illegible]
s__morganii	-0.037	0.586	-0.063	0.338	0.213	-0.063	0.603	0.56	0.565	-0.063	[illegible]
s__agglomerans	-0.009	0.818	-0.05	0.47	0.314	-0.05	-0.065	-0.079	0.78	-0.05	[illegible]
s__ananatis	0.058	0.389	-0.036	0.976	0.482	-0.036	-0.047	-0.057	0.562	-0.036	[illegible]
s__farmeri	-0.045	-0.077	-0.036	-0.043	-0.087	-0.036	-0.047	-0.057	-0.064	-0.036	[illegible]
s__segnis	-0.059	0.316	-0.086	0.386	0.841	-0.086	-0.109	-0.137	0.272	-0.086	[illegible]
s__influenzae	-0.045	-0.077	-0.036	0.183	-0.087	-0.036	-0.047	-0.057	-0.064	-0.036	[illegible]
s__parainfluenzae	-0.004	0.737	-0.075	0.627	0.459	-0.08	-0.103	-0.126	0.736	-0.079	[illegible]
s__guillouiae	-0.045	-0.077	-0.036	-0.043	-0.087	-0.036	-0.047	-0.057	-0.064	-0.036	[illegible]
s__alcaligenes	-0.088	-0.151	-0.07	0.026	-0.165	0.797	-0.087	-0.111	-0.127	-0.07	[illegible]
s__fragi	-0.059	-0.102	-0.047	-0.057	-0.115	-0.047	-0.062	-0.075	-0.086	-0.047	[illegible]
s__piscolens	-0.06	-0.102	-0.048	-0.041	-0.115	-0.048	-0.06	-0.076	-0.086	-0.048	[illegible]
s__muciniphila	-0.083	-0.142	-0.066	-0.08	-0.126	-0.025	0.846	0.792	-0.119	-0.066	[illegible]

											s__fetus					
0.982	-0.005	-0.087	-0.045	-0.045	-0.045	-0.056	-0.042	-0.045	0.058	-0.081	-0.045	-0.045	-0.045	-0.053	0.317	-0.073
-0.077	0.038	-0.15	-0.077	0.405	-0.077	-0.096	-0.003	0.705	0.389	0.173	-0.052	-0.077	0.405	-0.09	0.317	-0.126
1	0.001	-0.07	-0.036	-0.036	-0.036	-0.045	-0.019	-0.036	-0.036	-0.065	-0.043	-0.036	-0.036	-0.042	0.307	-0.059
-0.043	0.045	-0.084	-0.043	-0.043	-0.043	-0.054	-0.106	-0.043	0.976	-0.078	0.038	-0.043	-0.043	-0.05	0.274	-0.07
-0.087	-0.133	-0.165	-0.087	0.404	-0.048	-0.061	0.441	0.072	0.482	0.758	0.697	-0.052	0.404	-0.102	0.235	-0.132
-0.036	0.028	-0.07	1	-0.036	-0.036	-0.045	-0.089	-0.036	-0.036	-0.065	-0.043	-0.036	-0.036	-0.042	0.032	-0.059
-0.022	0.187	-0.091	-0.041	-0.044	0.981	-0.058	-0.111	-0.046	-0.047	-0.08	-0.053	-0.047	-0.044	-0.055	-0.125	0.352
0.159	0.218	-0.111	-0.057	-0.057	0.933	-0.071	-0.11	-0.057	-0.057	-0.103	-0.069	-0.057	-0.057	-0.067	-0.081	0.413
-0.064	0.073	-0.122	-0.064	-0.064	-0.064	-0.081	-0.159	0.558	0.562	-0.055	-0.022	-0.064	-0.064	-0.076	0.297	-0.104
-0.036	0.453	-0.07	-0.036	-0.036	-0.036	-0.045	-0.089	-0.036	-0.036	-0.065	-0.043	-0.036	-0.036	-0.042	-0.105	0.812
0.137	0.408	-0.158	-0.069	-0.084	-0.084	-0.106	-0.188	0.238	0.415	-0.09	-0.058	-0.084	-0.084	0.099	0.351	0.685
0.987	-0.012	-0.083	-0.043	-0.036	-0.043	-0.051	-0.032	-0.043	-0.043	-0.072	-0.049	-0.041	-0.036	-0.05	0.285	-0.069
0.956	-0.073	-0.116	-0.061	0.015	-0.069	-0.082	0.001	-0.069	-0.069	0.02	0.009	-0.065	0.015	0.071	0.432	-0.058
-0.103	0.201	-0.145	0.051	-0.103	0.868	-0.128	-0.18	0.071	0.068	-0.126	-0.11	-0.102	-0.103	0.137	0.089	0.448
-0.102	0.044	0.045	-0.056	-0.102	-0.102	-0.097	0.294	-0.102	-0.102	-0.084	-0.109	-0.08	-0.102	-0.054	-0.198	-0.143
-0.102	0.196	-0.134	0.517	-0.102	-0.103	-0.128	-0.049	0.059	0.062	-0.105	-0.109	-0.102	-0.102	-0.106	-0.055	-0.162
-0.048	0.022	-0.094	-0.048	-0.048	-0.048	-0.06	-0.119	-0.048	-0.048	-0.088	-0.059	-0.048	-0.048	-0.057	-0.129	-0.079
-0.064	0.507	0.256	-0.066	-0.067	-0.067	-0.084	0.562	-0.067	-0.067	-0.118	-0.082	-0.067	-0.067	-0.077	-0.181	-0.107
-0.068	-0.018	-0.134	-0.067	-0.069	0.701	-0.087	-0.145	-0.069	-0.069	-0.078	-0.084	-0.069	-0.069	0.098	-0.04	0.185
-0.145	0.052	-0.254	0.308	-0.155	-0.159	-0.175	-0.196	0.191	0.162	-0.168	-0.153	-0.141	-0.155	0.131	0.231	-0.128
-0.095	0.467	-0.141	-0.004	-0.12	-0.122	-0.144	-0.046	-0.036	-0.048	-0.129	-0.138	-0.115	-0.12	0.2	0.12	0.755
-0.06	0.093	-0.001	0.048	-0.095	-0.097	-0.098	0.114	-0.097	-0.097	-0.152	-0.112	-0.078	-0.095	-0.105	-0.23	-0.155
[illegible]																
