# Supplementary material for: Advanced glycation end products dietary restriction effects on bacterial gut microbiota in peritoneal dialysis patients; a randomized open label controlled trial
Source: PLoS One. 2017 Sep 20;12(9):e0184789. doi: 10.1371/journal.pone.0184789 (PMC5607175; doi:10.1371/journal.pone.0184789)
Supplement: S2 File — (DOC) [file pone.0184789.s009.doc]

# MSSM Protocol Template HRP-503a

| Instructions:   1. Prepare a document with the following sections. Note that, depending on the nature of your research, certain sections below may not be applicable. Indicate N/A as appropriate, explaining where possible. 2. For any items described in the sponsor’s protocol, grant application or other source documents submitted with the application, you may reference the title and page numbers of these documents rather than cutting and pasting into this document. **Do NOT refer to any derived documents, such as the Sample Consent document, or other internal documents required with the submission.** 3. If you reference page numbers, attach those pages to this protocol. 4. When you write a protocol, keep an electronic copy. You will need to modify this copy when making changes. |
| --- |

**Brief Summary of Research (250-400 words):**

Gut microbes can influence numerous aspects of human biology. Alterations in the function and composition of gut microbial flora (gut microbiota) have been linked to inflammatory bowel disease, chronic inflammation, dyslipidemia, diabetes mellitus, atopic disorders, cardiovascular disease, neoplasms, and obesity. Diet and other environmental factors have been found to alter the gut microbiome, and recent studies have shown that gut microbiota is altered in patients with end stage renal disease (ESRD) who are receiving renal replacement therapy (hemodialysis). However, little is known on whether the alteration in gut microbiota seen in renal patients is due to uremia. Several lines of evidence suggest that renal failure alters the chemical environment of the intestinal lumen which could impose a selective pressure on the growth of certain gut microbes, or is an epiphenomenal finding due to the complex confounding factors. This alteration in the gut microbiota of patients with renal failure significantly impacts the development of co-morbid conditions such as accelerated atherosclerosis, abnormal bone mineral metabolism, and chronic inflammation that are associated with renal failure. We hypothesize that the gut microbiota of patients with ESRD is highly diet dependant, and that changing the diet to a low advanced glycation end products (AGEs) diet will result in a better microbial composition and characteristics in the gut of patients with ESRD on peritoneal dialysis (PD).

1. Objectives:

*Research Question:*

- 1. How does a diet low or high in Advanced Glycation End products (AGEs) change the composition and activity of gut microbiota in patients with ESRD on PD?
  2. Will these changes in gut microbiota affect the serum levels of AGEs along with other inflammatory markers?
  3. Can a diet low in AGEs be associated with improvement in gut microbiota and therefore improve markers of chronic inflammation in ESRD patient?

The purpose of this study is to find out if a diet low in Advanced Glycation End products (Low-AGE diet) can improve the composition and diversity of gut bacteria in people with ESRD on PD.

1. Background

- A wide range of systemic diseases affects the kidney to cause chronic kidney disease (CKD) and end stage renal disease (ESRD), including autoimmune, metabolic and genetic diseases. Loss of kidney function in CKD/ESRD is associated with deleterious alterations in physiological and metabolic functions; uremic toxins are involved in the development of cardiovascular disease and progression of renal disease in CKD. While it is known that there is accumulation of bodily waste that is not excreted in individuals with renal failure, little has been done to delineate the production of uremic toxin, which are mainly produced by gut microbes and then absorbed from the gut into the bloodstream.
- Studies have shown that gut microbes can influence numerous aspects of human biology, and alterations in the function and composition of gut microbial flora (microbiota) play a major role in the pathogenesis of diverse human illnesses such as chronic inflammation, diabetes mellitus, and cardiovascular diseases. Gut microbes provide protection against pathogenic organisms, contribute to energy metabolism, serve a clear role in the development and modulation of the human gut immune system, and participate in nitrogen and micronutrient homeostasis by synthesizing amino acids and various vitamins. However, whether the composition of gut microbes is altered in humans with renal failure has not been clearly demonstrated. Furthermore, whether alterations in the gut microbiota due to renal failure contribute to development of co-morbid conditions associated with CKD has never been examined.
- By-products of protein and amino acid catabolism are known to contribute to medical conditions. For example, gut microbe-mediated protein catabolism produces P-cresol from tyrosine and phenylalanine , and indole from tryptophan which later is converted by the liver to indoxyl sulfate. P-cresol sulfate and indoxyl sulfate are considered uremic toxins because they contribute to adverse cardiac outcomes and serum levels of these compounds are elevated in patients with advanced kidney disease.
- There are several lines of evidence to suggest that the gut microbiota is likely altered in patients with CKD. It has been established that protein assimilation in the small intestine is impaired in CKD . As a consequence, an increased amount of dietary protein becomes available to the colon. The reduction in the ratio of available carbohydrate to protein in the colon may favor a shift from the more favorable saccharolytic to proteolytic fermentation pattern resulting in increased ammonia production. High levels of ammonia are responsible for elevated pH, which foster overgrowth of proteolytic species. In addition, colonic transit time is prolonged in CKD . As a result of increased transit time, a larger part of the colon becomes carbohydrate-deprived, which will induce an expansion of proteolytic species . A distinct gut microbiome with decreases in saccharolytic bacteria ( Lactobacillaceae and Prevotellaceae families) have been associated with CKD .
- Advanced Glycation End products (AGEs) are formed via the Maillard reaction, which consists of a complex network of non-enzymatic reactions involving the carbonyl groups of reducing sugars which react with the amino groups of proteins . AGEs are generated in vivo as a normal consequence of metabolism, but their formation is accelerated under conditions where blood glucose is chronically elevated such as poorly controlled diabetes . AGE formation is also increased in the presence of oxidative stress, which is frequently observed in individuals with the metabolic syndrome and diseases such as ESRD .
- AGEs affect mitochondrial function and results in the production of reactive oxygen species (ROS) . They also promotes the activation of several transcription factors including nuclear factor kappa-B (NF-κB), with subsequent upregulation of chemokines, such as MCP-1 and profibrogenic mediators such as TGFβ in addition to proinflammatory cytokines which are known to be involved in thrombogenesis, vascular inflammation and pathological angiogenesis. These AGE receptor- mediated events contribute to many of the long-term complications of diabetes and other diseases . AGE/AGE-receptor ligation also promotes overproduction of ROS which can then activate NF-κB, a key driver of inflammation .
- It is thought that certain dietary AGEs are largely undigested by human gut enzymes and eventually enter the colon, where they may act as a growth substrate for detrimental bacteria such as some Clostridium and Bacteroides species . Therefore it is conceivable that individuals who consume highly processed diets (which contain large quantities of AGEs) may adversely alter their colonic microbial composition, potentially enhancing their risk for the development of metabolic diseases such as obesity and type 2 diabetes .
- The purpose of this study is to find out if a diet low in Advanced Glycation End product (Low-AGE diet) can improve the composition and diversity of gut bacteria in patients with ESRD on PD.

1. Setting of the Human Research

This is a pilot study. All research procedures will be performed at Icahn School of Medicine at Mount Sinai. Potential study participants will be evaluated at Peritoneal Dialysis Unit:

Entrance:       1450 Madison Ave. and 99 street

(Klingenstein Clinical Center (KCC) Building)

Peritoneal Dialysis Unit (PDU): Annenberg Building, MC level, room 403 (Nursing Station)

Tel. 212-241-5537

*Patients with ESRD receiving PD at Icahn School of Medicine at Mount Sinai will be invited by their primary care nephrologist (Dr. Jaime Uribarri) to participate in the study.*

No involvement of any community advisory board for research conducted outside of MSSM.

1. Resources Available to Conduct the Human Research

We have a clinical research team made up of Dr. Jaime Uribarri, PI; Dr. John He, Co-Investigator; Dr. Rabi Yacoub, Co-Investigator; Dr. Melinda Nugent, Co-Investigator; Renata Pyzik, Senior Clinical Research Coordinator; and Dietitian, Elizabeth Tripp, RD, CDE.

At the beginning of this study, Standard Operating Procedure (SOP) will be established to ensure that all individuals involved in the study know the study procedures. Each member of the team will be adequately informed about the protocol, the investigational plans in reference to the AGE diet and their trial-related duties and functions.

1. Study Design
   1. Recruitment Methods

Potential participants will be identified and recruited from the Peritoneal Dialysis Unit. Patients with ESRD on PD and receiving treatment at Mount Sinai Hospital will be identified from the PD patient list.

All patients identified will receive a letter of invitation by mail informing them about the research project and inviting them to participate in the study. Two weeks after the invitation, the study team will contact the candidates by phone and explain the study protocol in detail, answer their questions and formally invite them to participate.

If the candidate agrees to participate in the study, a face-to-face interview will be scheduled. During this interview the investigators will explain the study design and intervention to the candidate, and if the candidate agrees to participate in the study, he/she will be asked to sign the informed consent form. Subjects will then be screened for inclusion/exclusion criteria and determined to be eligible or not for the study at that point. (Please refer to section d: Study timeline for details)

*We will also post the study on ClinicalTrials.gov website.*

- 1. Inclusion and Exclusion Criteria

Inclusion criteria:

- Age > 18 years.
- Patients with ESRD on PD.
- Patients are able to understand and give consent.
- Patients with estimated daily dietary AGE intake > 12 AGE Eq/day (12,000 kiloUnits/day) based on 3-day food records.

Exclusion criteria:

- History of diabetes mellitus.
- Patient on antibiotics in the last three months.
- Liver cirrhosis, and heart failure with EF < 30%.
- The use of chemotherapy, immunosuppressive medications, probiotics, and steroid in the last month.
- Oral iron supplementation in the last month.
- History of small or large intestine resection or small bowel obstruction.
  1. Number of Subjects

A minimum of 30 participants (15 in each group) is required. Based on previous experience, we expect a 20% drop out and extrapolate that an initial enrollment of 30 participants (15 in each group) will be sufficient to achieve at least 10 participants in each group at study end.

This is a pilot study. There are no prior studies evaluating the effects of dietary AGEs on gut microbiota in normal subjects or patients with different disease including kidney diseases. Thus the investigators cannot perform power analysis and sample size calculation. Based on our prior experience and recruitment rate and drop off noted when conducting a similar study we believe that 20 patients divided in two groups is a feasible and obtainable sample size.

Subjects will be divided in two groups:

- G1: 15 patients will continue their original diet.
- G2: 15 patients will be instructed to initiate a low AGE diet.
  1. Study Timelines

The duration anticipated to enroll all study subjects is 12 months after the IRB approval.

Participants will be enrolled in the study and followed for one month.

Study timeline will be divided to 4 steps.

All study visits will be held at Peritoneal Dialysis Unit (PDU):

Entrance:       1450 Madison Ave. and 99 street

(Klingenstein Clinical Center (KCC) Building)

Peritoneal Dialysis Unit : Annenberg Building, MC level, room 403 (Nursing Station)

Tel. 212-241-5537

**Step 1: Candidates identification:**

Invitation: All patients with ESRD on PD in the peritoneal dialysis unit will receive a letter of invitation and diet log sheet (forms attached).

Phone call 1 (15-30 minutes): Two weeks after sending the letters of invitation, the study team will contact the candidates and invite them to participate in the study. The investigators will explain in detail the hypothesis and objectives of the study, the methods of obtaining a dietary log and will answer all questions asked by the candidate. No HPI will be obtained in this phone call.

A screening and consent signing visit will be scheduled within 2 weeks after the phone call.

HIPPA waiver authorization to identify and contact the candidates is submitted (see attached).

Step 2: Screening and consent visit:

A face-to-face interview (60 minutes): This interview will be held within 1-2 weeks after “phone call 1.” During this interview, a detailed review of the study design, methods and goal will be provided and explained to the candidates. If the subject agrees to participate in the study, and to ensure compliance with HIPPA regulation, he/she will be asked to read/sign the consent form before obtaining any HPI. Signing the consent form does not mean that the subject is enrolled in the study. After signing the consent form, eligibility screen will be conducted and the subject will be enrolled in the study if eligible.

A review of medical history and medication to evaluate for eligibility will take place after signing the consent form.

Candidates will provide the dietary log and discuss it with the interviewer.

Candidates will be given consenting review/survey document (see attached) to answer to ensure full understanding of the study and acceptance to participate. All candidates will be provided with the appropriate tools to obtain the stool and urine samples (disposable: two ice packs, a box, two tubes, and 24 hour urine collection container).

Dietary evaluation: A dietitian will review the de-identified diet log and estimate the daily AGEs consumption.

Phone call 2: All candidates will be contacted within one week after the screening and consent signing visit to inform them about their eligibility to participate in the study. Candidates who are eligible from the medical history and medication perspective, and who consume a high AGE diet will be invited to participate in the study. A study first visit will be scheduled within 2 weeks and during their regular monthly dialysis visit. Candidates eligible to participate will be instructed to obtain the stool and urine samples and bring the samples to the study first visit.

**Step 2: first visit:**

Enrollment: At this point, candidates are officially enrolled in the study. . Patients on PD are seen by their primary nephrologist every month and blood tests are obtained monthly to evaluate dialysis efficacy and other parameters in this population (standard treatment for ESRD patients on PD).

The first visit will be scheduled to take place during one of the regular monthly dialysis visit, and the study blood samples will be obtained in addition to the blood samples obtained in the regular monthly visit.

Medical evaluation: This will take place during the PD monthly visit. Vital sign measurements and physical examination will be conducted as part of the standard of care, and reviewed by the investigators.

Diet evaluation: Candidates will bring detailed, accurate 3 day diet log for the nutritionist to evaluate.

Sample collection: Stool sample will be obtained and immediately stored in -80C at Annenberg building, floor 23 Dr. John He’s lab. A total of 45 ml (three tablespoons) will be obtained during this visit. 15 ml (one tablespoon) will be sent to the hospital laboratory to evaluate dialysis parameters as the standard of care. An extra blood sample [30 ml (two tablespoons)] will be obtained for the research study. Blood will be centrifuged to separate supernatant and mononuclear cells and both fractions will be immediately stored in -80C at Annenberg building, floor 23 (Dr. John He’s lab). 24 hour urine samples will be obtained during this meeting and immediately stored at -80C at Annenberg building, floor 23 Dr. John He’s lab (see paragraph f for details). Candidates will be given another set of sample collection tools (disposable: two ice packs, a box, two tubes, and urine collection container) to bring in the second visit.

Skin AGE measurement: Measurement of AGE levels in the skin will be done using an AGE reader. This AGE reader provides a non-invasive cardiovascular risk assessment by illuminating the skin of the forearm. The skin AGEs will emit a light that is detected by the AGE reader. The test will last about 10 seconds.

Randomization: Candidates will be randomly assigned to either continue the same diet [G1], or switch to low AGE diet [G2].

Diet education (30 minutes): The study dietitian will meet with both groups and instruct them in regards to food preparation and its content of AGE. Handouts will be given to both groups (see attached).

**Step 3: Phone calls follow up: (15 minutes)**

The study team will contact both groups twice weekly and take a brief diet evaluation to assess the AGE content of their diet and compliance with the protocol.

**Step 4: final evaluation:**

Visit: This visit will take place one month after the first visit (enrollment and randomization visit). This visit will be scheduled to take place during the regular monthly dialysis visit, and the study blood samples will be obtained in addition to the blood samples obtained in the regular monthly visit

Diet evaluation: Candidates will bring detailed, accurate 3 days diet log for the nutritionist to evaluate.

Sample collection: Stool sample will be obtained and immediately stored in -80C at Annenberg building, floor 23 Dr. John He’s lab. A total of 45 ml (three tablespoons) will be obtained during this visit. 15 ml (one tablespoon) will be sent to the hospital laboratory to evaluate dialysis parameters as the standard of care. An extra blood sample [30 ml (two tablespoons)] will be obtained for the research study. Blood will be centrifuged to separate supernatant and mononuclear cells and both fractions will be immediately stored in -80C at Annenberg building, floor 23 Dr. John He’s lab. 24 hour urine samples will be obtained during this meeting and immediately stored in -80C at Annenberg building, floor 23 Dr. John He’s lab.

Skin AGE measurement: Measurement of AGE levels in the skin will be done using an AGE reader. This AGE reader provides a non-invasive cardiovascular risk assessment by illuminating the skin of the forearm. The skin AGEs will emit a light that is detected by the AGE reader. The test will last about 10 seconds.

The expected duration of enrollment is **1** year.

The estimated date for the investigators to complete this study is 12/31/2016

- 1. Study Endpoints

Primary Endpoint: Change of gut microbiota after intervention.

Secondary Endpoint: Inflammatory and metabolic marker changes after intervention.

Safety endpoint: Candidates will be asked to either continue with their regular diet, or asked to cook their food in a different way, mainly boiling instead of frying/grilling. All cooking methods and measures to decrease AGE in diet are considered universally safe and are routinely recommended to the general population regardless of their medical conditions.

- 1. Procedures Involved in the Human Research

An initial phone interview will be conducted to invite potential study candidates to participate.

Face-to-face interviews will be performed to obtain consent and medical history as well as and provide training on recording dietary log. Stool, urine and blood samples will be obtained during both meetings.

Serum samples will be tested for levels of AGEs, IL-2, CRP, IL-10, creatinine, blood urea nitrogen, p-cresol, indoxyl sulfate, vitamin D and a global metabolite panel (the latter will be analyzed by non-targeted metabolite profiling platform combining ultrahigh performance liquid chromatography-tandem mass spectrometry optimized for basic species, optimized for acidic species, and gas chromatography/mass spectrometry). This metabolomic analysis will be performed by a third party (Metabolon). De-identified sample of one ml of serum will be sent to Metabolon® for metabolomic analysis.

AGE content, IL-2, CRP, IL-10, creatinine, blood urea nitrogen, p-cresol, indoxyl sulfate and vitamin D analysis will be done in Dr. He’s lab using ELISA, HPLC and other laboratory techniques as appropriate.

Metabolites in the urine will be analyzed by non-targeted metabolite profiling platform (the standard technique for metabolomic analysis). Urine will also be tested for AGEs,p-cresol, indoxyl sulfate using HPLC in Dr. He’s lab.

Stool will be used for comparisons of gut microbial composition by sequencing and comparing the V4 region of the 16S rRNA gene of bacterial DNA extracted from stool samples (De-identified samples will be sent to Dr. Jose Clemente’s lab, Mount Sinai Genetics and Genomic Sciences).

Measurement of AGE levels in the skin will be done using an AGE reader. This AGE reader provides a non-invasive cardiovascular risk assessment by illuminating the skin of the forearm. The skin AGEs will emit a light that is detected by the AGE reader. The test will last about 10 seconds.

At investigator’s lab blood will be centrifuged for separation of supernatant from peripheral mononuclear cells (PMNC). Supernatant will be tested for the AGEs: carboxymethyllysine (CML), methylglyoxal (MG), 8-isoprostane, insulin, leptin, and adiponectin. PMNC protein will be used to asses TNF-a, and intracellular AGEs. PMNC mRNA will be used to asses AGER1, RAGE and SIRT-1.

- 1. Specimen Banking

All samples (serum, urine and stool) will be stored and banked in the Annenberg building 23^rd^ floor, in -80 degree C temperature freezer. Samples will be stored until the study is concluded (estimate date 12/31/2016). Designated study person by PI will have access to the area where specimens are stored.

Samples will be given a unique letter code and no health protected information will be used in labeling the samples (As explained in details below in paragraph “h”).

Metabolon Inc. Durham NC. (Company responsible of metabolite analysis) will receive specimens labeled with the letter code. No protected health information will be submitted with the specimens. Regulatory agency will be permitted access the results of these analyses upon request. All requests will have to be approved by the study principle investigator.

Stool samples for microbiota study will be handled by Dr. Clemente’s lab.

- 1. Data Management and Confidentiality

Names and dates of birth will be stored in a file, and each patient will be assigned a letter code. Data collected will be stored in a separate file, using the assigned letter code as the identifier. Both files will be stored in a computerized database with a secure access and password available only to the investigators.

After initial processing of samples, a small portion of serum, stool and urine will be sent completely de-identified to Metabolon Inc. Durham NC. for metabolite analysis.

The results of their serum samples or urine samples will not be shared with the patients. No subject identifiers will be shared with Metabolon Inc. All data used in this study will be de-identified before being transferred to any person, organization, or database outside of Mount Sinai Medical Center. All patients will only be identified by the letter code (As mentioned above) on all study related materials in patient research folders and on the database. The log linking ID numbers to patient identities will be kept in a password protected and encrypted file on a Mount Sinai networked computer. The data will be kept for at least 2 years after the completion of the study. When transmitting data, only de- identified data will be used. All identifiable patient information will be stored on Mount Sinai’s protected server. All paper copies of forms and patient health information will be stored in a locked room at Mount Sinai Hospital.

Data will be analyzed using SPSS statistical program, for categorical values chi-square test will be used, for non categorical values analysis of variance (ANOVA) will be used.

Gut microbiota will be analyzed by sequencing of V4 region of 16S rRNA. Sequencing data will be analyzed using Qiime program (Dr. Clemente’s lab).

- 1. Provisions to Monitor the Data to Ensure the Safety of subjects

**Part I** describes the safety monitoring activities that will be undertaken in during the study. This should be completed for all studies that require more than the basic minimum DSMP.

**Part II** describes Data and Safety Monitoring Committees or Boards and should be completed when one is needed for the DSMP

**Part III** describes requests for alteration of some or all of the reporting requirements for adverse events. This may be part of a larger DSMP but may also be completed by investigators using the basic minimum DSMP as described in the Data and Safety Monitoring Guidance.

**Part I: Elements of a Data and Safety Monitoring Plan**

1. List the name(s) of the individual(s) at MSSM who will be responsible for data and safety monitoring of this study. For each individual, indicate their role, name, title, and department information. The Principal Investigator may be the only monitor of a study.

If the qualifications of an individual to serve as a monitor are not contained in the PPHS application, they must be added to the DSMP either as a narrative description or as a CV.

**MSSM Principal Monitor:**

Check One: PI: Team Member:  Independent:

Last Name: Uribarri

First Name: Jaime

Academic Title: Professor of Medicine

Department: Medicine

Mailing Address: MSSM, One Gustave L. Levy Place, Box 1147

New York, NY 10029

Phone: 212-241-1887

Fax: 212-369-9339

E-mail: jaime.uribarri@mssm.edu

**MSSM Additional Monitor:**

Check One: PI:  Team Member:  Independent:

Last Name: Yacoub

First Name: Rabi

Academic Title: Renal Fellow

Department: Medicine

Mailing Address: MSSM, One Gustave L. Levy Place, Box 1147

New York, NY 10029

Phone: 716-609-8855

Fax: 212- 369-9339

E-mail: rabi.yacoub@mountsinai.org

**MSSM Additional Monitor:**

Check One: PI:  Team Member:  Independent:

Last Name: Winston

First Name: Jonathan

Academic Title: Professor of Medicine

Department: Medicine

Mailing Address: MSSM, One Gustave L. Levy Place, Box 1243,

New York, NY 10029

Phone: 212 241-8131

Fax: 212 241-0389

E-mail: jonathan.winston@mssm.edu

Drs. Uribarri and Yacoub will be responsible for data and safety monitoring for this study on a daily basis. Dr. Jonathan Winston will perform an independent monitoring in our study every three months.

2. Justify your choice of principal monitor in terms of the assessed risk to the research subject‘s health and wellbeing. In high risk studies when the principal monitor is independent of the study staff, indicate the individual’s credentials, relationship to the PI, and the rationale for selection.

This is an open-label, low-risk dietary intervention involving adult subjects with peritoneal dialysis, in a single clinical site over a period of about 1 month, and with one research team. Therefore, an independent DSMB is not warranted. Drs. Jaime Uribarri and Rabi Yacoub will be evaluating each and all of these patients on a regular basis to determine any potential adverse events. Dr. Jonathan Winston will monitor independently the study, and will start after the enrollment of the first five participants and every 3 months onwards.

3. List the specific items that will be monitored for safety (e.g., adverse events, subject compliance with the protocol, drop outs, etc.).

***Data monitoring***

*The following elements will be monitored:*

- *Is recruitment proceeding as expected?*
- *Do cohort characteristics match the exclusion/inclusion criteria*
- *Any deviations from protocol*
- *Comment on timeliness, accuracy and confidentiality of all information both in study documents and database*
- *Missing data points*
- *Dropout rates*

***Safety monitoring***

*The following elements will be monitored:*

- *Serious and unanticipated adverse events*
- *The totality of adverse events including expected and less than serious*
- *Reasons for subjects withdrawing from the study*
- *Changes in nutritional parameters from baseline*
- *Changes in concomitant medications*

4. Indicate the frequency at which **ACCUMULATED** safety and data information (items listed in number 3 above and interim analysis of efficacy outcomes) will be reviewed by the monitor(s) or the Data Monitoring Committee (DMC). Although this information must be reviewed at least annually, the higher the study risks, the more frequently reviews must be scheduled.

Monitoring will take place at every visit or following any serious adverse event until the completion of the study. Cumulative safety and data monitoring will be reviewed semiannually. Independent monitor will review cumulative data and safety semiannually.

Semiannually we will create a summary of safety to be reviewed by the Independent Monitor, Dr. Jonathan Winston, and to be sent to the MSSM IRB with an annual progress report.

5. Where applicable, describe rules which will guide interruption or alteration of the study design.

Participation of a person will be terminated in cases of noncompliance with study diet and design. The participation of candidates who fail to answer the follow up phone calls for 2 consecutive weeks, or report the nonadherence to diet for more than one week will be terminated.

6. Where applicable, indicate dose selection procedures that will be used to minimize toxicity.

N/A. This is a dietary intervention. No dose selection is possible.

7. List any specialized grading system that will be used to evaluate adverse events (e.g., National Cancer Institute Common Toxicity Criteria).

N/A.

8. Describe procedures that will be used to assure data accuracy and completeness.

This is a study involving a small number of subjects for about 1-month period by an experienced research team (Dr. J. Uribarri, Dr. Rabi Yacoub, Dr. Melinda Nugent, research coordinator(s) and study dietitian(s)), which will pay personal attention to each participant.

9. Should a temporary or permanent suspension of your study occur, in addition to the PPHS, to whom (NIH, FDA, sponsor, IRB) will you report the occurrence?

Any such situation will be immediately reported to the IRB, and PDU.

**Part II. Data Monitoring Committee/Data Safety Monitoring Board (DMC/DSMB)**

When appropriate, attach a description of the DMC. Provide the number of members of the DMC, their names and area of professional expertise. DMC reports must be made available to the local PI and the MSSM PPHS. The report need not contain specifics of the study or data, but there must be assurance that subject safety is not being compromised and that the results of treatment do not warrant early termination of the study.

There will be a weekly clinical study meeting for the duration of study. The agenda for this meeting will include discussion of subject recruitment, problems with the current participants, review of available data, missing data, patient drop outs, evaluation of the nutritional status of patients and follow up of laboratory test results. This meeting will be conducted by the PI and co-investigator(s) and attended by the study dietitian(s) and the study coordinator(s). The PI will be responsible for monitoring the literature regarding the study topics and bring to this forum any new information that has implications for this study. The need for any potential changes in the protocol suggested by this new information will be discussed by the research team.

**Part III. Alteration of Reporting Requirements**

For some studies it may be appropriate to request a waiver from reporting requirements for some or all adverse events and/or serious adverse events as required by MSSM policy.

If you are requesting an alteration to standard AE reporting requirements, select either A or B and complete that section: N/A

**A:** Requesting a waiver of all AEs/SAEs/Death on Protocol reporting requirements (note: unanticipated problems must still be reported). The justification for this request is: N/A _

**B:** Requesting another type of alteration:

The alteration to MSSM AE/UP reporting requirements that is being requested is: N/A

The justification for the request is: N/A

Data integrity, storage and analysis will be assured by the PI.

In case of loss of data containing identifiable information, this will be reported to the IRB using FORM HRP-224 within 5 business days.

Requests by regulatory agencies to inspect the study sites may be made. The clinical investigator agrees to allow inspectors from regulatory agencies to review records and to assist the inspectors in their duties, if requested.

Source documents are the physician’s or hospital’s patient records maintained at the study site.

- 1. Withdrawal of Subjects

Subjects may stop taking part in this research study at any time without any penalty. This will not affect their ability to receive medical care at Mount Sinai or to receive any benefits to which they are otherwise entitled.

If subjects decide to stop being in the research study, they can contact the Principal Investigator or the research staff.

They may also withdraw their permission for the use and disclosure of any of their protected information for research, but they must do so in writing to the Principal Investigator at the address on the first page. Even if they withdraw their permission, the Principal Investigator for the research study may still use the information that was already collected if that information is necessary to complete the research study. Their health information may still be used or shared after they withdraw their authorization if a subject should have an adverse event (a bad effect) from participating in the research study.

Withdrawal without subject’s consent: The study doctor, the sponsor or the institution may stop a subject’s involvement in this research study at any time without their consent. This may be because the research study is being stopped, the instructions of the study team have not been followed, the investigator believes it is in their best interest, or for any other reason. If specimens or data have been stored as part of the research study, they too can be destroyed without their consent.

1. Risks to Subjects

There are no known risks associated with low AGE diet. If participants are randomized to the high AGE diet (usual diet), they are not going to change their diet, and then the risks of maintaining that diet remain the same.

Qualified staff will draw blood using standard sterile equipment. Some people may experience a small amount of pain. On rare occasions, minor bruising may occur. These effects will self-resolve within hours or days. Occasionally, people feel faint or faint after blood draws.

Participants will be asked to bring a 24-hour urine and stool collection for 2 visits, which may create some inconvenience.

There are no risks associated with the AGE reader which only uses a light source to illuminate AGEs in the skin.

We cannot identify any physical or social risks for study participants.

There is a slight concern about possible lost of earnings during the study visit; however, the subject will determine and decide if participating in the study is more important/beneficial then earning wages.

There always exists the potential for loss of private information; however, there are procedures in place to minimize this risk.

1. Provisions for Research Related Injury

All subjects who will experience any side effect that is thought to be secondary to their participation in this study will be provided with all the medical or psychological resources they might need. Subjects with any kind of side effect should contact the study team.

1. Potential Benefits to Subjects

Participants are not expected to get any benefit from taking part in this research.  Although there is no direct benefit to participants in this study, we hope the results will help us to better understand the influence of dietary factors in the progression of the kidney disease. Therefore, we believe the risk/benefit ratio of this research favors the benefits.

1. Provisions to Protect the Privacy Interests of Subjects

All discussions with patients will be conducted in a private area away from distractions, in order to maintain patient privacy. The patient will have control of/be responsible for scheduling their appointments at a time that is convenient for them. In order to make the subject feel at ease, study personnel will be accessible, available, and receptive to questions and concerns raised by the patient about study-related issues. It is acceptable and appropriate for members of the research team to approach the prospective participant about research because they are a subject that fits the inclusion/exclusion criteria for the study and are open to discussing participation in a research protocol.

1. Economic Impact on Subjects

There will be no charge to the patient for their participation in this study. The study visits will be provided at no charge to the patient or their insurance company.

1. Payment to Subjects

Participants in this study will be compensated for study visits, each visit $50. A total of $100 for both study visits.

1. Consent Process

A waiver of consent for identifying potential subjects via the PD clinic records, and to contact the candidates via mail and phone calls will be obtained. Candidates will need to give a verbal approval and accept to continue and participate in the phone call when called for the first time.

After explaining the study thoroughly, if the candidate agrees to participate, informed consent will be obtained. Consent will be obtained in office. The setting in which the consent is requested and obtained will be one in which the potential subject can consider the request as an autonomous individual, free from time constraints or a sense of obligation or dependency. Potential subjects will be given the opportunity to review the consent document, discuss it with whomever they like, digest the information that has been presented to them and be able to make an informed decision, without any element of coercion. Subjects who sign the consent form and fulfill the inclusion/exclusion criteria are considered enrolled to the study. A translator will be present to Spanish speaking individuals to answer all questions and explain the study procedures.

SOP HRP-090 Informed Consent Process for Research will be used.

Children, cognitively impaired adults and vulnerable subjects will not be enrolled.

Spanish speaking subjects will be given the consent form in Spanish.

1. Process to Document Consent in Writing

The standard PPHS consent template is used for this study. All subjects consented for the study will sign a copy of the informed consent document and will be offered a copy of the document to take home for their own records.

1. Vulnerable Populations

| Include | Exclude | Vulnerable Population Type |
| --- | --- | --- |
|  | X | Adults unable to consent |
|  | X | Individuals who are not yet adults (e.g. infants, children, teenagers) |
|  | X | Wards of the State (e.g. foster children) |
|  | X | Pregnant women |
|  | X | Prisoners |

1. Multi-Site Human Research (Coordinating Center)

N/A.

1. Community-Based Participatory Research

N/A.

1. Sharing of Results with Subjects

Subjects have the right to see and make copies of their PHI. However, as a subject in this study, they will be agreeing to not see or copy some or all of their PHI until the sponsor has completed all work related to the study, at which point they may ask to see their records.

1. IRB Review History

This is initial submission to the IRB.

1. Control of Drugs, Biologics, or Devices

*Note: The IDS has its own forms that must be completed and a review process that must be followed before the IDS representative will sign off on Appendix B for submission to the PPHS.*

N/A.

**References:**

1. De Smet R, Glorieux G, Hsu C, Vanholder R. p-cresol and uric acid: two old uremic toxins revisited. Kidney international Supplement. 1997;62:S8-11. PubMed PMID: 9350670.

2. Deguchi T, Ohtsuki S, Otagiri M, Takanaga H, Asaba H, Mori S, et al. Major role of organic anion transporter 3 in the transport of indoxyl sulfate in the kidney. Kidney international. 2002;61(5):1760-8. doi: 10.1046/j.1523-1755.2002.00318.x. PubMed PMID: 11967025.

3. Bammens B, Evenepoel P, Keuleers H, Verbeke K, Vanrenterghem Y. Free serum concentrations of the protein-bound retention solute p-cresol predict mortality in hemodialysis patients. Kidney international. 2006;69(6):1081-7. doi: 10.1038/sj.ki.5000115. PubMed PMID: 16421516.

4. Meijers BK, Claes K, Bammens B, de Loor H, Viaene L, Verbeke K, et al. p-Cresol and cardiovascular risk in mild-to-moderate kidney disease. Clinical journal of the American Society of Nephrology : CJASN. 2010;5(7):1182-9. doi: 10.2215/CJN.07971109. PubMed PMID: 20430946; PubMed Central PMCID: PMC2893077.

5. Vanholder R, De Smet R, Hsu C, Vogeleere P, Ringoir S. Uremic toxicity: the middle molecule hypothesis revisited. Seminars in nephrology. 1994;14(3):205-18. PubMed PMID: 8036355.

6. Bammens B, Verbeke K, Vanrenterghem Y, Evenepoel P. Evidence for impaired assimilation of protein in chronic renal failure. Kidney international. 2003;64(6):2196-203. Epub 2003/11/25. doi: 10.1046/j.1523-1755.2003.00314.x. PubMed PMID: 14633143.

7. Wu MJ, Chang CS, Cheng CH, Chen CH, Lee WC, Hsu YH, et al. Colonic transit time in long-term dialysis patients. American journal of kidney diseases : the official journal of the National Kidney Foundation. 2004;44(2):322-7. Epub 2004/07/21. PubMed PMID: 15264191.

8. Lefebvre HP, Ferre JP, Watson AD, Brown CA, Serthelon JP, Laroute V, et al. Small bowel motility and colonic transit are altered in dogs with moderate renal failure. American journal of physiology Regulatory, integrative and comparative physiology. 2001;281(1):R230-8. Epub 2001/06/19. PubMed PMID: 11404298.

9. Cummings JH, Hill MJ, Bone ES, Branch WJ, Jenkins DJ. The effect of meat protein and dietary fiber on colonic function and metabolism. II. Bacterial metabolites in feces and urine. The American journal of clinical nutrition. 1979;32(10):2094-101. Epub 1979/10/01. PubMed PMID: 484528.

10. Cummings JH, Hill MJ, Jivraj T, Houston H, Branch WJ, Jenkins DJ. The effect of meat protein and dietary fiber on colonic function and metabolism. I. Changes in bowel habit, bile acid excretion, and calcium absorption. The American journal of clinical nutrition. 1979;32(10):2086-93. Epub 1979/10/01. PubMed PMID: 484527.

11. Vaziri ND, Wong J, Pahl M, Piceno YM, Yuan J, DeSantis TZ, et al. Chronic kidney disease alters intestinal microbial flora. Kidney Int. 2013;83(2):308-15. doi: 10.1038/ki.2012.345. PubMed PMID: 22992469.

12. Henle T. Protein-bound advanced glycation endproducts (AGEs) as bioactive amino acid derivatives in foods. Amino acids. 2005;29(4):313-22. doi: 10.1007/s00726-005-0200-2. PubMed PMID: 15997413.

13. Brownlee M. Biochemistry and molecular cell biology of diabetic complications. Nature. 2001;414(6865):813-20. doi: 10.1038/414813a. PubMed PMID: 11742414.

14. Folli F, Corradi D, Fanti P, Davalli A, Paez A, Giaccari A, et al. The role of oxidative stress in the pathogenesis of type 2 diabetes mellitus micro- and macrovascular complications: avenues for a mechanistic-based therapeutic approach. Current diabetes reviews. 2011;7(5):313-24. PubMed PMID: 21838680.

15. Uribarri J, Peppa M, Cai W, Goldberg T, Lu M, He C, et al. Restriction of dietary glycotoxins reduces excessive advanced glycation end products in renal failure patients. Journal of the American Society of Nephrology : JASN. 2003;14(3):728-31. PubMed PMID: 12595509.

16. Rosca MG, Mustata TG, Kinter MT, Ozdemir AM, Kern TS, Szweda LI, et al. Glycation of mitochondrial proteins from diabetic rat kidney is associated with excess superoxide formation. American journal of physiology Renal physiology. 2005;289(2):F420-30. doi: 10.1152/ajprenal.00415.2004. PubMed PMID: 15814529.

17. Schmidt AM, Hori O, Chen JX, Li JF, Crandall J, Zhang J, et al. Advanced glycation endproducts interacting with their endothelial receptor induce expression of vascular cell adhesion molecule-1 (VCAM-1) in cultured human endothelial cells and in mice. A potential mechanism for the accelerated vasculopathy of diabetes. The Journal of clinical investigation. 1995;96(3):1395-403. doi: 10.1172/JCI118175. PubMed PMID: 7544803; PubMed Central PMCID: PMC185762.

18. Morita M, Yano S, Yamaguchi T, Sugimoto T. Advanced glycation end products-induced reactive oxygen species generation is partly through NF-kappa B activation in human aortic endothelial cells. Journal of diabetes and its complications. 2013;27(1):11-5. doi: 10.1016/j.jdiacomp.2012.07.006. PubMed PMID: 22944044.

19. Mills DJ, Tuohy KM, Booth J, Buck M, Crabbe MJ, Gibson GR, et al. Dietary glycated protein modulates the colonic microbiota towards a more detrimental composition in ulcerative colitis patients and non-ulcerative colitis subjects. Journal of applied microbiology. 2008;105(3):706-14. doi: 10.1111/j.1365-2672.2008.03783.x. PubMed PMID: 18399977.

20. Nakamura YK, Omaye ST. Metabolic diseases and pro- and prebiotics: Mechanistic insights. Nutrition & metabolism. 2012;9(1):60. doi: 10.1186/1743-7075-9-60. PubMed PMID: 22713169; PubMed Central PMCID: PMC3464869.
